# *Stenotrophomonas maltophilia*: A Gram-Negative Bacterium Useful for Transformations of Flavanone and Chalcone

**DOI:** 10.3390/molecules22111830

**Published:** 2017-10-27

**Authors:** Edyta Kostrzewa-Susłow, Monika Dymarska, Urszula Guzik, Danuta Wojcieszyńska, Tomasz Janeczko

**Affiliations:** 1 Department of Chemistry, Faculty of Biotechnology and Food Science, Wrocław University of Environmental and Life Sciences, Norwida 25, 50-375 Wrocław, Poland; monika.dymarska@gmail.com (M.D.); janeczko13@interia.pl (T.J.); 2 Department of Biochemistry, Faculty of Biology and Environmental Protection, University of Silesia in Katowice, Jagiellonska 28, 40-032 Katowice, Poland; urszula.guzik@us.edu.pl (U.G.); danuta.wojcieszynska@us.edu.pl (D.W.)

**Keywords:** flavanone, chalcone, dihydrochalcone, biotransformation, *Stenotrophomonas maltophilia*

## Abstract

A group of flavones, isoflavones, flavanones, and chalcones was subjected to small-scale biotransformation studies with the Gram-negative *Stenotrophomonas maltophilia* KB2 strain in order to evaluate the capability of this strain to transform flavonoid compounds and to investigate the relationship between compound structure and transformation type. The tested strain transformed flavanones and chalcones. The main type of transformation of compounds with a flavanone moiety was central heterocyclic C ring cleavage, leading to chalcone and dihydrochalcone structures, whereas chalcones underwent reduction to dihydrochalcones and cyclisation to a benzo-γ-pyrone moiety. Substrates with a C-2–C-3 double bond (flavones and isoflavones) were not transformed by *Stenotrophomonas maltophilia* KB2.

## 1. Introduction

For transformations of flavonoid compounds, enzymatic systems of filamentous fungi [1,2,3,4], yeasts [5,6], bacteria [7,8,9], higher plants [10], and isolated enzymes are used [11]. The main aim of these transformations is to improve the biological properties of flavonoids [12,13,14], increase their antioxidant properties [15,16], and obtain derivatives with high optical purity [17].

Biotransformation strategies for the production of flavonoids have attracted considerable interest because they allow the generation of novel flavonoids. The main reactions during biotransformation are hydroxylation and *O*-methylation, *O*-demethylation, glycosylation, deglycosylation, dehydrogenation, hydrogenation, C ring cleavage of the benzo-γ-pyrone system, cyclization, and carbonyl reduction [18]. The biocatalysts of hydroxylation, in addition to cytochrome P450 monooxygenases, are laccases and peroxidases [19].

Many redox biotransformations of flavonoids are conducted using whole-cell systems, in which cofactor regeneration systems already exist [20]. Cytochrome P450 monooxygenases are often used as whole-cell systems because of the need for cofactor recycling. Some of the interesting biocatalytic applications of laccases and phenol oxidases (and peroxidases) also involve coupling reactions [21]. Makris and Rossiter reported cleavage of the flavonoid skeleton during the oxidation of quercetin, morin, and rutin with mushroom polyphenol oxidase and horseradish peroxidase [22], while Mejias et al. reported the oxidation of quercetin and rutin by horseradish or soybean peroxidase, leading to polymerization products [23].

For the biotransformations described in this paper, we used the Gram-negative *S. maltophilia* KB2 strain, which is known to be capable of the biodegradation of a wide range of aromatic compounds [24,25,26]. There are known transformations of a non-substituted flavanone, catalyzed by the enzymatic system of the Gram-positive *Rhodococcus* sp. DSM 364 strain, which resulted in heterocyclic C-ring [7] cleavage and the reduction of the α,β-unsaturated bond in chalcone, leading to respective dihydrochalcone [27].

The isolation, purification, and analysis of genetic and biochemical properties revealed that the *S. maltophilia* KB2 strain contains enzymes belonging to oxygenases: phenol monooxygenase [28] and catechol 2,3-dioxygenase [29]. Phenol monooxygenase is the enzyme that takes part in the key step of phenolic compound decomposition. It is responsible for the hydroxylation of a phenolic ring so that two hydroxyl groups are located either *ortho* or *para* to each other. Such a dihydroxylated intermediate is necessary for the next step of aromatic compound decomposition.

Phenol monooxygenase from *S. maltophilia* KB2 is an FAD-dependent, 34-fold purified oxygenase, with a molecular mass of 34 kDa. NADH is an electron donor in the reactions of hydroxylation. The maximum activity of the enzyme occurs at pH 7.2 and a temperature of 30 °C. The activity of the monooxygenase of the KB2 strain is associated with the cytochrome system [28].

Apart from monooxygenases, the second group of enzymes that is crucial for the degradation of aromatic compounds is dioxygenases, which cleave an aromatic ring and incorporate dioxygen into a substrate. The *S. maltophilia* KB2 strain in the presence of phenol demonstrates high activity of catechol 2,3-dioxygenase. Catechol 2,3-dioxygenase is a typical mesophilic enzyme and shows maximum activity at pH 7.6 and 30 °C. The maximum activity is equal to 61.37 U/mg of the total protein, which indicates that this enzyme is an exceptionally active biocatalyst [29].

The aim of our study was to determine how the bacteria, which are known to decompose phenolic compounds, will transform aromatic compounds of higher molecular masses, namely, derivatives of flavone, isoflavone, flavanone, and chalcone. The enzymatic system of the *S. maltophilia* KB2 strain, apart from the cleaving properties of intradiol and extradiol dioxygenases, was promising for obtaining the desired dihydroxyl flavonoid derivatives.

The selection study, carried out in a group of 21 flavonoid substrates, demonstrated that the *S. maltophilia* KB2 strain did not perform a total degradation (to phenol, phenylacetic acid, or phenylpropionic acid derivatives [9,30]) of any of the tested flavonoids. The observed transformations included cyclisation, reduction, hydroxylation, demethylation, and heterocyclic flavanone C-ring cleavage.

## 2. Results and Discussion

The initial selection study (described in Section 3.4) was performed with the following flavonoid compounds: 6-hydroxyflavone, 6-methoxyflavone, 7-hydroxyflavone, 7-methoxyflavone, 6-methylflavone, 7,4′-dihydroxyisoflavone (daidzein), flavanone, 6-hydroxyflavanone, 6-methoxyflavanone, 7-hydroxyflavanone, 7-methoxyflavanone, 2′-methoxyflavanone, 3′-hydroxyflavanone, 3′-methoxyflavanone, 4′-methoxyflavanone, 5,7-dimethoxyflavanone, 5,7,4′-trihydroxyflavanone (naringenin), 2′-hydroxy-2-methoxychalcone, 2′,3-dihydroxychalcone, 2′-hydroxy-3-methoxychalcone, and 2′-hydroxy-4-methoxychalcone. The tests were conducted according to the principles of ‘green chemistry’, using microtiter plates, which require minimal amounts of cultivation media, substrates, and solvents. The selection study proved that flavones and isoflavones are not transformed by the *S. maltophilia* KB2 strain. For biotransformations on a larger scale, six of the substrates were selected. Each of them gave two or more transformation products. The selected substrates included: 7-methoxyflavanone, 5,7-dimethoxyflavanone, 2′-hydroxy-3-methoxychalcone, flavanone, 6-methoxyflavanone, and 2′,3-dihydroxychalcone. The results for the first three flavonoid substrates are presented in this article, whereas the biotransformations of the next three will be described in our next report.

### 2.1. Biotransformations of 7-Methoxyflavanone (***1***)

**Figure 1:**
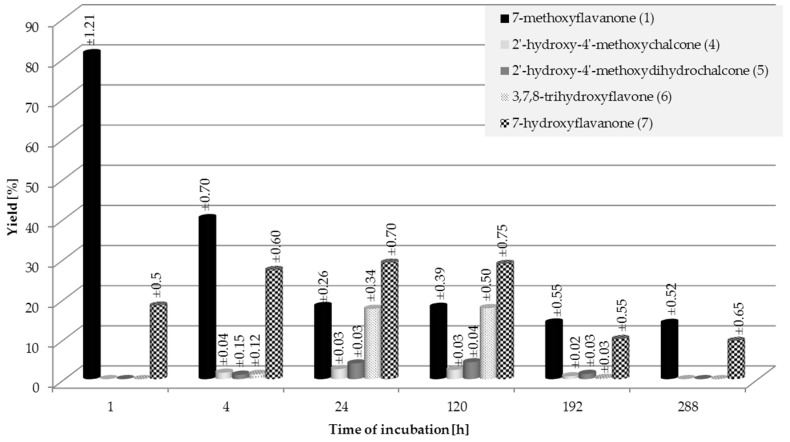
Biotransformation of 7-methoxyflavanone (**1**) in *S. maltophilia* KB2 culture; the yield (%) of the products was determined by HPLC.

Transformations of racemic 7-methoxyflavanone (**1**) in the culture of *S. maltophilia* KB2 strain started in the first hour of incubation. We observed the process of demethylation. The yield of obtained 7-hydroxyflavanone (**7**) rose from 18.3% in the first hour to 27.2% in the fourth hour of biotransformation (Figure 1). In the fourth hour of the process, small amounts of the other three biotransformation products appeared: 2′-hydroxy-4′-methoxychalcone (**4**) (1.6%), 2′-hydroxy-4′-methoxydihydrochalcone (**5**) (1.1%), and 3,7,8-trihydroxyflavone (**6**) (1.3%). In the first 24 h, the amounts of all four products increased, whereas between the fifth and the twelfth day their content dropped drastically (Figure 1). On the twelfth day, we observed 14% of the unreacted substrate (**1**) and 9.5% of 7-hydroxyflavanone (**7**) in the reaction mixture.

**Scheme 1:**
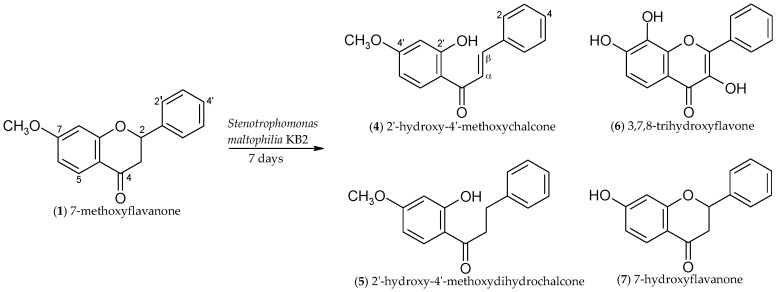
Microbial transformation of 7-methoxyflavanone (**1**) in *S. maltophilia* KB2 culture.

In order to isolate the four products and to carry out their spectroscopic analysis, a seven-day biotransformation of 7-methoxyflavanone (**1**) was carried out on a larger scale. The products were isolated with the yields as follows: 2′-hydroxy-4′-methoxychalcone (**4**) (2.3%), 2′-hydroxy-4′-methoxydihydrochalcone (**5**) (3.7%), 3,7,8-trihydroxyflavone (**6**) (16.2%), and (±)-7-hydroxyflavanone (**7**) (28.4%) (Scheme 1). 17.3% of the unreacted substrate (**1**) was left in the post-reaction mixture.

In the proton nuclear magnetic resonance (^1^H-NMR) spectrum of 2′-hydroxy-4′-methoxychalcone (**4**), two one-proton doublets appear at δ = 7.89 ppm and δ = 7.59 ppm. They have identical coupling constants (*J* = 15.5 Hz) and are characteristic for chalcones (H-α and H-β). At δ = 13.43 ppm, there is a singlet of the hydroxyl group at C-2′ in the chalcone (**4**), which is not observed in the spectrum of the substrate—7-methoxyflavanone (**1**). Comparing the ^1^H-NMR spectrum of the second product, 2′-hydroxy-4′-methoxydihydrochalcone (**5**), with the substrate (7-methoxyflavanone), two new two-proton multiplets appear at δ 3.24 and 3.06 ppm, ascribed to Hα and Hβ and characteristic for dihydrochalcones. However, there are not two doublets of doublets of H-3ax (δ = 3.05 ppm, *J*_3ax,3eq_ = 16.9 Hz, *J*_3ax,2_ = 13.4 Hz) and H-3eq (δ = 2.84 ppm, *J*_3eq,3ax_ = 16.9 Hz, *J*_3eq,2_ = 2.9 Hz) and a one-proton doublet of doublets of H-2 at δ = 5.47 ppm (*J*_2,3ax_ = 13.4 Hz, *J*_2,3eq_ = 2.9 Hz), which is typical of a flavanone moiety. In the ^1^H-NMR spectrum of product **5**, there is an additional singlet at δ = 12.79 ppm, which is ascribed to the C-2′ hydroxyl group. In the ^1^H-NMR spectrum of 3,7,8-trihydroxyflavone (**6**), we can see the protons of hydroxyl groups at C-3 (δ = 6.13 ppm) and C-8 (δ = 6.09 ppm), which are not present in the spectrum of 7-methoxyflavanone (**1**). However, there is no doublet of doublets of H-2 at δ = 5.47 ppm and no two doublets of doublets of H-3ax and H-3eq (δ = 3.02 ppm and δ = 2.85), which means that dehydration at C-2 and C-3 took place, leading to the double bond. Additionally, there is no doublet of H-8 (δ = 6.51 ppm) visible for substrate (**1**) because it is replaced by the signal of the hydroxyl group at C-8. The ^1^H-NMR spectrum of **6** also lacks the 3-proton singlet of 7-OCH_3_, observed for **1**. The presence of substituents at C-3 and C-8 in the biotransformation product (3,7,8-trihydroxyflavone) is also confirmed in the ^13^C-NMR spectrum by the movement of the C-3 signal from δ = 44.3 ppm for substrate **1** to δ = 135.9 ppm for product **6** and the signal of C-8 from δ=100.9 ppm for 7-methoxyflavanone (**1**) to δ = 166.2 ppm for product **6**.

### 2.2. Biotransformations of 5,7-Dimethoxyflavanone (***2***)

**Figure 2:**
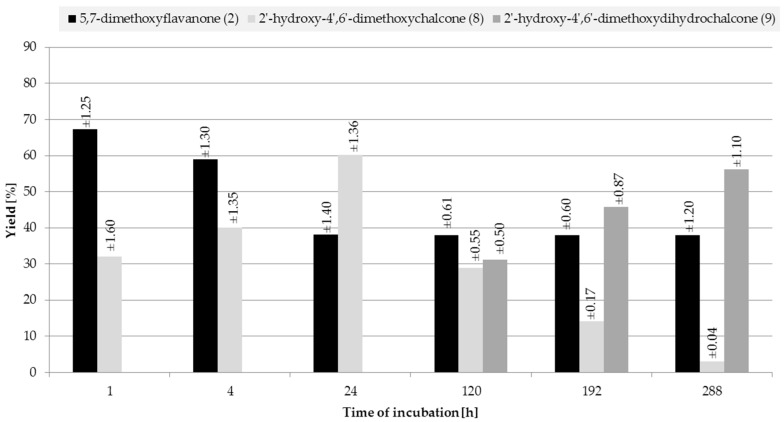
Biotransformation of 5,7-dimethoxyflavanone (**2**) in *S. maltophilia* KB2 culture; the yield (%) of the products was determined by HPLC.

Monitoring the course of biotransformation of (±)-5,7-dimethoxyflavanone (**2**) we observed that, from the first to the 24th hour of the reaction, there was only the product of flavanone heterocyclic ring C cleavage present: 2′-hydroxy-4′,6′-dimethoxychalcone (**8**). On the first day, product **8** was observed with 60.2% yield and 62% of substrate **2** conversion. On the fifth day, the second product appeared: 2′-hydroxy-4′,6′-dimethoxydihydrochalcone (**9**). From the fifth to the twelfth day of the reaction, a constant level of unreacted substrate **2** was observed (38%), whereas the yield of 2′-hydroxy-4′,6′-dimethoxydihydrochalcone (**9**) increased from 31.1% (fifth day) to 56.1% (twelfth day), along with a decrease in the amount of 2′-hydroxy-4′,6′-dimethoxychalcone (**8**) (Figure 2).

**Scheme 2:**
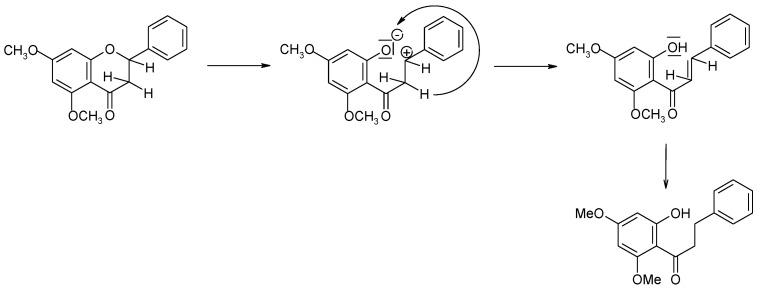
Probable transformation of 5,7-dimethoxyflavanone (**2**) in *S. maltophilia* KB2 culture.

During the biotransformation of (±)-5,7-dimethoxyflavanone (**2**), no considerable loss of the substrate or the products caused by biodegradation was observed. The results allowed the most probable course of the transformation of (±)-5,7-dimethoxyflavanone (**2**) to be presented. The first step is a cleavage of the heterocyclic C ring, resulting in the formation of a very stable benzylic carbocation. Next, one of the hydrogen atoms is attracted by the lone electron pair of oxygen, which leads to formation of a hydroxyl group at C-2′ and therefore to a chalcone structure. Then the chalcone undergoes enzymatic reduction to dihydrochalcone (**9**) (Scheme 2).

**Scheme 3:**
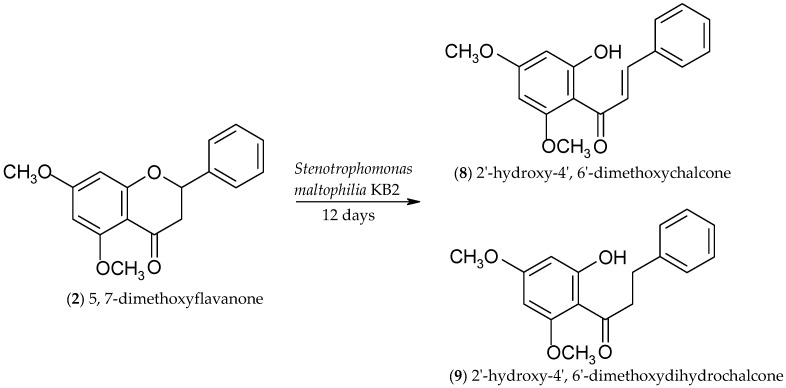
Microbial transformation of 5,7-dimethoxyflavanone (**2**) in *S. maltophilia* KB2 culture.

A 12-day biotransformation of (±)-5,7-dimethoxyflavanone (**2**) carried out on a larger scale allowed us to isolate both products, 2′-hydroxy-4′,6′-dimethoxychalcone (**8**) and 2′-hydroxy-4′,6′-dimethoxydihydrochalcone (**9**), with yields of 2.7% and 54.1%, respectively (Scheme 3). 31% of the unreacted substrate remained in the reaction mixture.

Similarly to the transformation of 7-methoxyflavanone (**1**), one of the products of the biotransformation of 5,7-dimethoxyflavanone (**2**) is a compound with a chalcone structure: 2′-hydroxy-4′,6′-dimethoxychalcone (**8**). The characteristic change in the ^1^H-NMR spectrum of product **8** compared to substrate (**2**) is the lack of two doublets of doublets due to H-3ax (δ = 3.02 ppm) and H-3eq (δ = 2.80 ppm), observed for 5,7-dimethoxyflavanone (**2**). Also, there is no doublet of doublet of H-2 (δ = 5.41 ppm), whereas two new doublets of Hα (δ = 7.90 ppm) and Hβ (δ= 7.79 ppm) appear with the same coupling constant (*J* = 15.6 Hz) and a singlet at δ = 14.28 ppm, due to a hydroxyl group at C-2′ of the chalcone (**8**). The second product obtained in the biotransformation of 5,7-dimethoxyflavanone (**2**) is 2′-hydroxy-4′,6′-dimethoxydihydrochalcone (**9**). In the spectrum of this product there are no signals of H-3ax (δ = 3.02 ppm) and H-3eq (δ = 2.80 ppm) (two doublets of doublets), and also there is no doublet of doublet of H-2 (δ = 5.41 ppm), which is characteristic of flavanones. However, two multiplets appeared at δ = 3.35 ppm and δ = 3.03 ppm due to H-α and H-β, respectively. Additionally, a signal at δ = 14.05 ppm is observed, which is due to the 2′-OH hydroxyl group formed after flavanone C-ring cleavage.

### 2.3. Biotransformation of 2′-Hydroxy-3-methoxychalcone (***3***)

**Figure 3:**
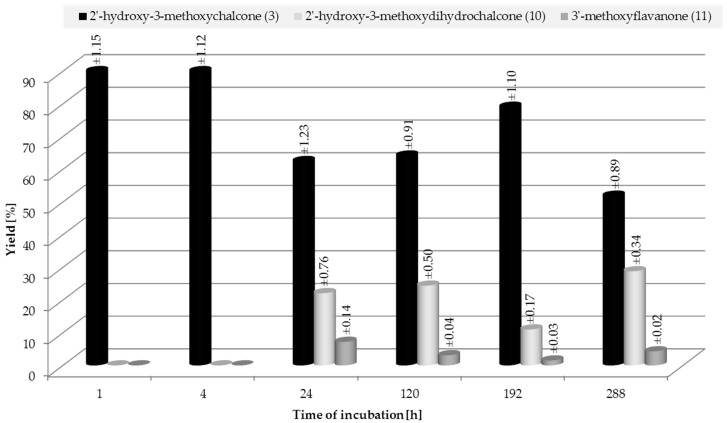
Biotransformation of 2′-hydroxy-3-methoxychalcone (**3**) in *S. maltophilia* KB2 culture; the yields (%) of the products were determined by HPLC.

Monitoring the course of the biotransformation of 2′-hydroxy-3-methoxychalcone (**3**), we observed that, from the first to the twelfth hour of the reaction, the amounts of the two biotransformation products, 2′-hydroxy-3-methoxydihydrochalcone (**10**) and 3′-methoxyflavanone (**11**), were changing dynamically (Figure 3).

**Scheme 4:**
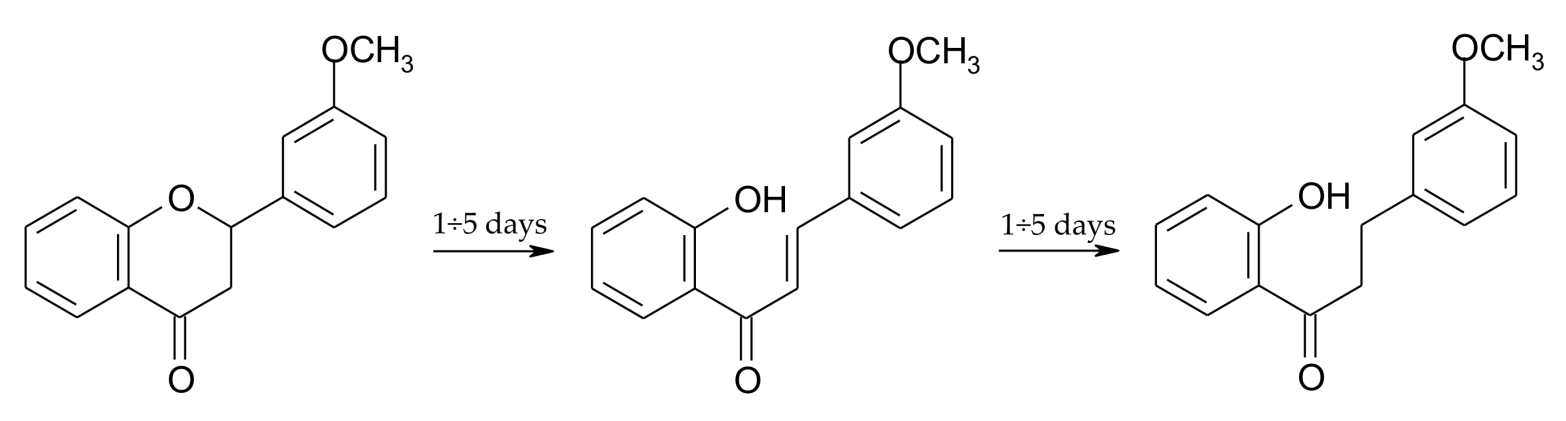
Dynamic transformations occurring between the first and the fifth day of the biotransformation of 2′-hydroxy-3-methoxychalcone (**3**) in *S. maltophilia* KB2 culture.

On the first day of the transformation, we observed 22.1% of **10** and 7.2% of **11**, and there was 62.5% of unreacted substrate **3** in the reaction mixture. From the first to the fifth day, we noted a small increase in the amount of chalcone (**3**) and dihydrochalcone (**10**) (each about 2%), along with a decrease (about 4%) in the amount of flavanone (**11**) (Figure 3) (Scheme 4).

**Scheme 5:**
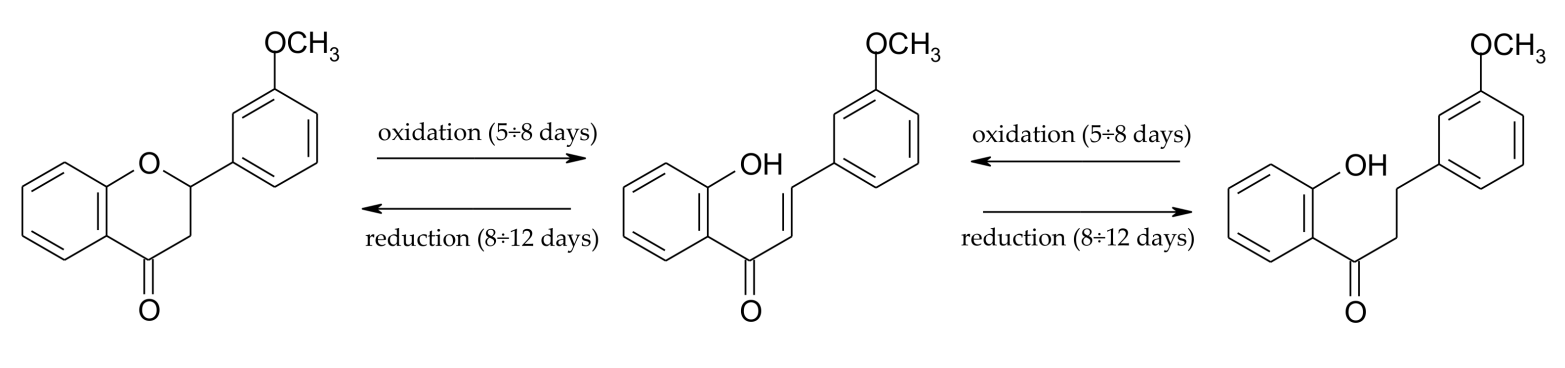
Dynamic transformations occurring from the fifth to the eighth and from the eighth to the twelfth day of the biotransformation of 2′-hydroxy-3-methoxychalcone (**3**) in *S. maltophilia* KB2 culture.

From the fifth to the eight day of the process, the amount of chalcone (**3**) increased by 15%, which was accompanied by a proportional decrease in the amount of dihydrochalcone (**10**) and flavanone (**11**) (Scheme 5). However, from the eighth to the twelfth day the amount of chalcone (**3**) fell by 27%, along with a rise in the amount of 2′-hydroxy-3-methoxydihydrochalcone (**10**) by 17.8% and 3′-methoxyflavanone (**11**) by 2.8% (Scheme 5).

**Scheme 6:**
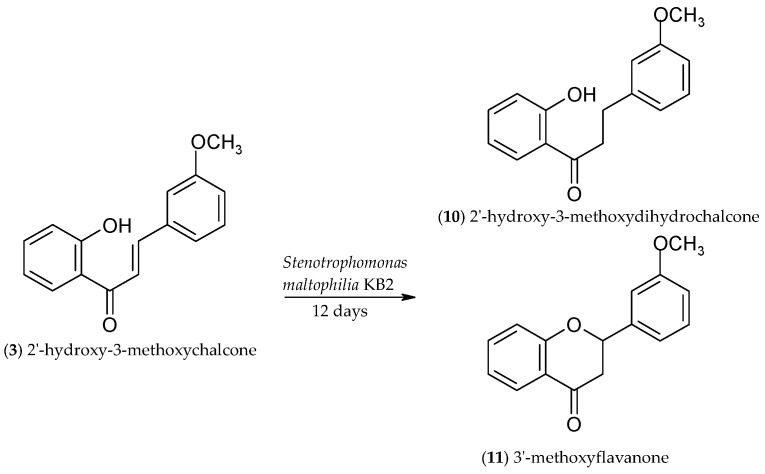
Microbial transformation of 2′-hydroxy-3-methoxychalcone (**3**) in *S. maltophilia* KB2 culture.

After a 12-h biotransformation of 2′-hydroxy-3-methoxychalcone (**3**) performed on a larger scale, we isolated 36.1% of 2′-hydroxy-3-methoxydihydrochalcone (**10**) and 15.2% of 3′-methoxyflavanone (**11**) (Scheme 6). 37.8% of unreacted substrate **3** remained in the reaction mixture.

In the ^1^H-NMR spectrum of 2′-hydroxy-3-methoxydihydrochalcone (**10**) and other products with dihydrochalcone structures, there are two characteristic multiplets due to protons at C-α and C-β: a two-proton signal at δ = 3.33 ppm (H-α) and a two-proton signal at δ = 3.05 ppm (H-β).

The second product obtained in the biotransformation of **10** was found to be 3′-methoxyflavanone (**11**). In the ^1^H-NMR of **11**, there is no signal of the hydroxyl group at δ = 12.79 ppm present in the spectrum of substrate **10,** and also lacking are, characteristic of chalcones, two doublets at δ = 7.89 ppm and δ = 7.65 ppm due to H-α and H-β. However, we observed, typically for flavanones, doublets of doublets at δ = 3.08 ppm and δ = 2.90 ppm due to 3ax and 3eq protons, respectively, and a signal of H-2 as a doublet of doublets at δ = 5.46 ppm.

## 3. Materials and Methods

### 3.1. Analysis

The course of the microbial transformation was monitored by thin layer chromatography (TLC) (SiO_2_, DC Alufolien Kieselgel 60 F_254_, Merck, Darmstadt, Germany). Chromatograms were developed using the following developing systems: hexane:ethyl acetate (7:3), dichloromethane:ethyl acetate (1:1), and toluene:diethyl ether (4:1).

The biotransformation products were separated and purified using preparative TLC glass plates coated with silica gel (Analtech TLC Uniplates). To develop the chromatograms, an eluent consisting of hexane and ethyl acetate (5:1) was used. The NMR spectra were recorded with a DRX 600 MHz Bruker spectrometer (Bruker, Billerica, MA, USA). Mass spectra were obtained using high resolution electrospray ionization (ESI^+^-MS) (Waters LCT Premier XE mass spectrometer, Milford, MA, USA). The optical rotation was measured with a Jasco P-2000-Na automatic polarimeter (ABL&E-JASCO, Kraków, Poland). The pH measurements were carried out with a PH-100ATC pH-meter (Voltcraft, Colchester, UK).

The HPLC analyses were performed with a Waters 2690 instrument equipped with a Waters 996 photodiode array detector, using an ODS 2 column (4.6 × 250 mm, Waters, Milford, MA, USA) and a Guard- Pak Inserts μBondapak C18 pre-column. The separation conditions were as follows: gradient elution, using 80% acetonitrile in 4.5% formic acid solution (eluent A) and 4.5% formic acid (eluent B); flow, 1 mL/min; detection wavelength 280 nm; program: 0 to 7 min, 10% A 90% B; 7 to 10 min, 50% A 50% B; 10 to 13 min, 60% A 40% B; 13 to 15 min, 70% A 30% B; 15 to 20 min 80% A 20% B; 20 to 30 min 90% A 10% B; 30 to 40 min, 100% A.

The enantiomeric excess values were determined using a Chiralpak AD-H HPLC column with diameters 4.6 × 250 mm (Diacel, Bratislava, Slovakia), with hexane:isopropanol (9:1) as the eluent (isocratic resolution).

### 3.2. Materials

The substrates for biotransformation were as follows: 7-methoxyflavanone (**1**) was purchased from Sigma Chemical Company (St. Louis, MO, USA); 5,7-dimethoxyflavanone (**2**) was obtained following the method by Yeom et al. [31]; and 2′-hydroxy-3-methoxychalcone (**3**) was obtained following the method by Yadav et al. [32].

#### 3.2.1. 7-Methoxyflavanone (**1**)

C_16_H_14_O_3_; Rt 20.54 min (HPLC); purity 97% (HPLC); [α]${}_{589}^{20}$ = 0, (c = 0.95, CH_3_OH); ^1^H-NMR, see Table 1; ^13^C-NMR, see Table 2. High-resolution electrospray ionisation mass spectrometry (HRESI-MS) [M + H^+^] was calculated/found (*m*/*z* 255.1020/255.1003) [33,34] (Supplementary Materials).

#### 3.2.2. 5,7-Dimethoxyflavanone (**2**)

C_17_H_16_O_4_; Rt 17.80 min (HPLC); purity 99% (HPLC); [α]${}_{589}^{20}$ = 0, (c = 1.00, THF); ^1^H-NMR, see Table 1; ^13^C-NMR, see Table 2. HRESI-MS [M+H^+^] was calculated/found) (*m*/*z* 285.1222/285.1213).

#### 3.2.3. 2′-Hydroxy-3-methoxychalcone (**3**)

C_16_H_14_O_3_; Rt 25.69 min (HPLC); purity 98% (HPLC); ^1^H-NMR, see Table 3; ^13^C-NMR, see Table 2. HRESI-MS [M + H^+^] was calculated/found (*m*/*z* 255.1150/255.1144).

### 3.3. Microorganism

The *Stenotrophomonas maltophilia* KB2 strain was obtained from the Department of Biochemistry of the University of Silesia (Faculty of Biology and Environmental Protection) (Poland) [24]. The strain is deposited and available in the VTT culture collection (Finland) under collection number E-113197.

### 3.4. Biotransformations

The *S. maltophilia* KB2 strain used for the biotransfomations was cultivated in PCM culture medium consisting of bacto peptone 10 g/L, casein hydrolysate 2 g/L, yeast extract 2 g/L, NaCl 6 g/L, glucose 20 g/L, and agar 15 g/L. Specified amounts of the culture medium ingredients were dissolved in distilled water and placed on microtiter plates (4 mL per well). Sterilization was carried out in an autoclave at 121 °C for 15 min. Our study started with the preparation of a sufficient amount of the bacterial culture for inoculation. For this reason, the bacteria were transferred from the slants to two 500 mL Erlenmeyer flasks, each containing 150 mL of the sterilized cultivation medium, and the flasks were incubated at 28 °C for 24 h on shakers. Then the flasks were stored at 4 °C.

The initial selection tests were carried out on microtiter plates (Duetz-System, EnzyScreen B.V., Heemstede, The Netherlands). The culture medium on the plates (4 mL per well) was inoculated with 100 µL of the inoculation culture and incubated at 28 °C for 24 h on a shaker. Then 0.4 mg of the same substrate dissolved in 0.1 mL of THF was added to each well. After one hour, four hours, one day, five, eight, and 12 days, the whole content of a well was taken out and extracted with ethyl acetate; the organic layer was separated, dried over anhydrous MgSO₄, and filtered into a test tube; and the solvent was evaporated off. Control cultivation with no substrate was also performed. The residue was analyzed by thin layer chromatography (TLC). Quantitative analyses of the mixtures were performed by means of HPLC. Calibration curves for the quantitative analyses were prepared using isolated and purified biotransformation products as standards.

Biotransformations on a preparative scale were carried out in 500 mL Erlenmeyer flasks containing 200 mL of a sterile PCM culture medium. Each of the flasks was inoculated with 100 µL of the inoculation culture and incubated at 28 °C for 48 h on a shaker. Then flavonoid substrates dissolved in 0.5 ml of THF were added: three flasks to each 20 mg of the substrate. Additionally, one control flask with no substrate was prepared. The flasks with 7-methoxyflavanone were incubated for seven days, and, for the rest of the substrates, the incubation was continued for 12 days at 28 °C on shakers.

After the relevant incubation time, the mixtures were extracted with ethyl acetate (3 × 200 mL), dried (MgSO_4_), and concentrated in vacuo. The transformation products were separated by means of preparative TLC plates, and the structures of the products were established by means of spectroscopic methods: 1D ^1^H-NMR, ^13^C-NMR, and also two-dimensional NMR spectroscopy (homonuclear correlation spectroscopy (COSY), heteronuclear single quantum coherence spectroscopy (HSQC)).

The physical and spectral data of the products obtained are presented below (Table 1, Table 2 and Table 3) (Supplementary materials).

#### 3.4.1. 2′-Hydroxy-4′-methoxychalcone (**4**)

C_16_H_14_O_3_; Rt 19.35 min (HPLC); purity 98% (HPLC); ^1^H-NMR, see Table 3; ^13^C-NMR, see Table 2. HRESI-MS [M + H^+^] was calculated/found (*m*/*z* 255.1160/255.1155).

#### 3.4.2. 2’-Hydroxy-4’-methoxydihydrochalcone (**5**)

C_16_H_16_O_3_; Rt 14.67 min (HPLC); purity 99% (HPLC); ^1^H-NMR, see Table 3; ^13^C-NMR, see Table 2. HRESI-MS [M + H^+^] was calculated/found (*m*/*z* 257.1265/257.1259).

#### 3.4.3. 3,7,8-Trihydroxyflavone (**6**)

C_15_H_10_O_5_; Rt 14.24 min (HPLC); purity 98% (HPLC); ^1^H-NMR see Table 1; ^13^C-NMR see Table 2. HRESI-MS [M + H^+^] (calculated/found) (*m*/*z* 271.0660/271.0654).

#### 3.4.4. 7-Hydroxyflavanone (**7**)

C_15_H_12_O_3_; Rt 16.06 min (HPLC); purity 97% (HPLC); [α]${}_{589}^{20}$ = 0, (c = 0.40, THF); ^1^H-NMR, see Table 1; ^13^C-NMRm see Table 2. HRESI-MS [M + H^+^] was calculated/found) (*m*/*z* 241.0866/241.0859).

#### 3.4.5. 2′-Hydroxy-4′,6′-dimethoxychalcone (**8**)

C_17_H_16_O_4_; Rt 25.18 min (HPLC); purity 98% (HPLC); ^1^H-NMR, see Table 3; ^13^C-NMR, see Table 2. HRESI-MS [M + H^+^] was calculated/found) (*m*/*z* 285.1219/285.1215).

#### 3.4.6. 2′-Hydroxy-4′,6′-dimethoxydihydrochalcone (**9**)

C_17_H_18_O_4_; Rt 13.56 min (HPLC); purity 99% (HPLC); ^1^H-NMR, see Table 3; ^13^C-NMR, see Table 2. HRESI-MS [M + H^+^] was calculated/found (*m*/*z* 287.1558/287.1552).

#### 3.4.7. 2′-Hydroxy-3-methoxydihydrochalcone (**10**)

C_16_H_16_O_3_; Rt 16.65 min (HPLC); purity 99% (HPLC); ^1^H-NMR, see Table 3; ^13^C-NMR, see Table 2. HRESI-MS [M + H^+^] was calculated/found (*m*/*z* 257.1120/257.1115).

#### 3.4.8. 3′-methoxyflavanone (**11**)

C_16_H_14_O_3_; Rt 19.52 min (HPLC); purity 99% (HPLC); [α]${}_{589}^{20}$ = 0, (c = 1.00, THF); ^1^H-NMR, see Table 1; ^13^C-NMR, see Table 2. HRESI-MS [M+H^+^] was calculated/found (*m*/*z* 255.1165/255.1159).

## 4. Conclusions

Compounds with flavanone and chalcone structures are transformed by the *S. maltophilia* KB2 strain. The main type of transformation of flavanone compounds is a cleavage of the heterocyclic C-ring, leading to chalcone and dihydrochalcone structures, whereas the major type of transformation of chalcones is a reduction to a dihydrochalcone structure and a cyclisation, resulting in the formation of a benzo-γ-pyrone moiety. Flavonoids with a C-2, C-3 double bond are not transformed by *S. maltophilia* KB2. The unsaturated bond in the C ring makes the molecule more rigid, and this may be a steric hindrance for the enzyme catalytic center. The *S. maltophilia* KB2 strain isolated for the bioremediation of phenol and its derivatives does not perform a total degradation of either the substrates chosen for biotransformation or the flavonoid compounds.

## Figures and Tables

**Table 1:** ^1^H-NMR chemical shifts (δ) of flavanones (**1**, **2**, **7**, **11**) and flavone (**6**) (Supplementary Materials).

Proton	Compound				
	1 ^a^	2 ^a^	7 ^a^	11 ^b^	6 ^b^
H-2	5.47 (dd) (*J*_2,3ax_ = 13.4, *J*_2,3eq_ = 2.9)	5.41 (dd) (*J*_2,3ax_ = 13.2, *J*_2,3eq_ = 2.6)	5.46 (dd) (*J*_2,3ax_ = 12.9, *J*_2,3eq_ = 3.0)	5.46 (dd) (*J*_2,3ax_ = 13.3, *J*_2,3eq_ = 2.8)	-
H-3	H-3_ax_ 3.05 (dd) (*J*_3ax,3eq_ = 16.9, *J*_3ax,2_ = 13.4) H-3_eq_ 2.84 (dd) (*J*_3eq,3ax_ = 16.9, *J*_3eq,2_ = 2.9)	H-3_ax_ 3.02 (dd) (*J*_3ax,3eq_ = 16.5, *J*_3ax,2_ = 13.3) H-3_eq_ 2.80 (dd) (*J*_3eq,3ax_ = 16.5, *J*_3eq,2_ = 2.8)	H-3_ax_ 2.95 (dd) (*J*_3ax,3eq_ = 16.9, *J*_3ax,2_ = 12.8) H-3_eq_ 2.69 (dd) (*J*_3eq,3ax_ = 16.9, *J*_3eq,2_ = 3.1)	H-3_ax_ 3.08 (dd) (*J*_3ax,3eq_ = 16.8, *J*_3ax,2_ = 13.4) H-3_eq_ 2.90 (dd) (*J*_3eq,3ax_ = 16.5, *J*_3eq,2_ = 2.9)	-
H-5	7.87 (d) (*J*_5,6_ = 8.8)	-	7.72 (d) (*J*_5.6_ = 8.7)	7.94 (dd) (*J* = 8.2, *J* = 1.6)	7.07 (d) (*J* = 8.1)
H-6	6.63 (dd) (*J*_6,5_ = 8.8, *J*_6,8_ = 2.4)	6.16 (d) (*J*_6,8_ = 2.0)	6.46 (dd) (*J*_6.5_ = 8.7, *J*_6.8_ = 2.2)	7.06 (m)	6.81 (d) (*J* = 8.2)
H-7	-	-	-	7.52 (m)	-
H-8	6.51 (d) (*J*_8,6_ = 2.4)	6.10 (d) (*J*_8,6_ = 1.9)	6.35 (d) (*J*_8.6_ = 2.2)	7.06 (m)	-
H-2’	7.48 (d) (*J*_2′,3′_ = 6.4)	7.46 (d) (*J* = 7.5)	7.49 (d) (*J*_2’,3’_ = 7.6)	7.06 (m)	7.23 (d) (*J* = 7.5)
H-3’	7.39 (m)	7.42 (t) (*J* = 7.5)	7.38 (m)	-	7.35 (m)
H-4’	7.39 (m)	7.37 (t) (*J* = 7.2)	7.32 (m)	6.92 (m)	7.29 (m)
H-5’	7.39 (m)	7.42 (t) (*J* = 7.5)	7.38 (m)	7.35 (t) (*J* = 8.2)	7.35 (m)
H-6’	7.48 (d) (*J*_6′,5′_ = 6.4)	7.46 (d) (*J* = 7.5)	7.49 (d) (*J*_6′,5′_ = 7.6)	7.06 (m)	7.23 (d) (*J* = 7.5)
3-OH	-	-	-	-	6.13 (s)
8-OH	-	-	-	-	6.09 (s)
7-OH	-	-	9.70 (s)	-	-
7-OCH_3_	3.84 (s)	3.82 (s)	-	-	-
5-OCH_3_	-	3.90 (s)	-	-	-
3’-OCH_3_	-	-	-	3.85 (s)	-

^a^ Solvent THF-d_8_, ^b^ Solvent CDCl_3_.

**Table 2:** ^13^C-NMR chemical shifts (δ) of flavonoids **1** to **11** (Supplementary Materials).

Carbon	Flavonoids										
	1 ^a^	2 ^a^	3 ^b^	4 ^b^	5 ^b^	6 ^b^	7 ^a^	8 ^b^	9 ^b^	10 ^b^	11 ^b^
C-1	-	-	136.1	135.0	141.0	-	-	135.7	142.1	142.5	-
C-2	80.2	79.4	113.9	128.6	128.5	169.5	81.0	128.7	128.7	114.4	79.6
C-3	44.5	45.7	160.2	129.1	128.7	135.9	45.4	129.0	128.6	159.9	44.9
C-4	190.7	189.3	116.8	130.7	126.4	170.3	193.1	130.2	126.1	111.7	192.1
C-5	129.1	162.4	130.2	129.1	128.7	130.4	129.6	129.0	128.6	129.8	127.2
C-6	110.4	93.3	121.4	128.6	128.5	116.1	111.4	128.7	128.7	120.9	118.5
C-7	166.4	166.1	-	-	-	165.1	166.9	-	-	-	136.4
C-8	101.1	93.7	-	-	-	166.2	103.7	-	-	-	118.3
C-9	163.7	165.1	-	-	-	165.1	165.4	-	-	-	160.1
C-10	115.0	106.1	-	-	-	116.1	115.4	-	-	-	121.1
C-1′	139.0	138.9	120.2	-	113.6	-	140.7	-	141.9	119.4	140.4
C-2′	126.3	126.2	163.8	167.0	165.6	128.5	127.2	168.6	167.8	162.6	121.8
C-3′	129.0	128.9	129.8	107.9	107.8	129.1	129.4	93.9	93.8	118.7	161.6
C-4′	128.9	128.8	119.0	166.4	166.2	127.6	129.2	107.8	162.9	136.5	114.2
C-5′	129.0	128.9	136.6	101.2	101.1	129.1	129.4	91.4	91.0	119.1	130.1
C-6′	126.3	126.2	118.8	131.4	131.6	128.5	127.2	111.9	166.1	130.0	112.0
C-α	-	-	145.6	144.7	39.8	-	-	127.7	45.8	40.1	-
C-β	-	-	120.6	120.6	30.5	-	-	142.5	30.8	30.2	-
C=O	-	-	193.9	-	203.7	-	-	202.1	204.8	205.5	-
3-OCH_3_	-	-	55.6	-	-	-	-	-	-	55.3	-
5-OCH_3_	-	56.3	-	-	-	-	-	-	-	-	-
7-OCH_3_	55.8	55.7	-	-	-	-	-	-	-	-	-
3′-OCH_3_	-	-	-	-	-	-	-	-	-	-	55.5
4′-OCH_3_	-	-	-	55.8	55.7	-	-	55.8	55.8	-	-
6′-OCH_3_	-	-	-	-	-	-	-	56.0	55.7	-	-

^a^ Solvent THF-d_8_; ^b^ Solvent CDCl_3._

**Table 3:** ^1^H-NMR chemical shifts (δ) of chalcones (**3**, **4**, **8**) and dihydrochalcones (**5**, **9**, **10**) in CDCl_3_ (Supplementary Materials).

Proton	Chalcones			Dihydrochalcones		
	3	4	8	5	9	10
H-α	7.89 (d) (*J* = 15.5)	7.89 (d) (*J* = 15.5)	7.90 (d) (*J* = 15.6)	3.24 (m)	3.32 (m)	3.33 (m)
H-β	7.65 (d) (*J* = 15.5)	7.59 (d) (*J* = 15.5)	7.79 (d) (*J* = 15.6)	3.06 (m)	3.03 (m)	3.05 (m)
H-3’	7.93 (d) (*J* = 8.0)	6.51 (m)	6.12 (s)	6.41 (d) (*J* = 2.3)	6.10 (d) (*J*_3′,5′_ *=* 2.3)	6.99 (d) (*J* = 8.4)
H-4’	6.96 (t) (*J* = 7.6)	-	-	-	-	7.47 (t) (*J* = 7.7)
H-5’	7.51 (t) (*J* = 7.8)	6.49 (m)	5.97 (s)	6.43 (m)	5.92 (d) (*J*_5′,3′_ = 2.2)	6.88 (t) (*J* = 7.6)
H-6’	7.04 (d) (*J* = 8.4)	7.84 (d) (*J* = 8.8)	-	7.64 (d) (*J* = 8.6)	-	7.75 (d) (*J* = 7.8)
H-2	7.18 (s)	7.66 (m)	7.61 (d) (*J* = 7.4)	7.25 (d) (*J*_2,3(6,5)_ = 7.5)	7.24 (d) (*J*_2,3(6,5)_ = 7.7)	6.80 (s)
H-3	-	7.43 (m)	7.41 (m)	7.30 (t) (*J* = 7.5)	7.30 (t) (*J* = 7.5)	-
H-4	7.00 (dd) (*J* = 8.2, *J* = 2.0)	7.43 (m)	7.41 (m)	7.22 (t) (*J* = 7.4)	7.20 (t) (*J* = 7.2)	6.77 (dd) (*J* = 8.3, *J* = 2.1)
H-5	7.36 (t) (*J* = 7.9)	7.43 (m)	7.41 (m)	7.30 (t) (*J* = 7.5)	7.30 (t) (*J* = 7.5)	7.23 (t) (*J* = 7.9)
H-6	7.27 (d) (*J* = 7.7)	7.66 (m)	7.61 (d) (*J* = 7.4)	7.25 (d) (*J*_2,3(6,5)_ = 7.5)	7.24 (d) (*J*_2,3(6,5)_ = 7.7)	6.84 (d) (*J* = 7.5)
2′-OH	12.79 (s)	13.43 (s)	14.28 (s)	12.79 (s)	14.05 (s)	12.29 (s)
3-OCH_3_	3.88 (s)	-	-	-	-	3.80(s)
4′-OCH_3_	-	3.87 (s)	3.84 (s)	3.84 (s)	3.82 (s)	-
6′-OCH_3_	-	-	3.93 (s)	-	3.84 (s)	-

## Supplementary Materials

Supplementary Materials are available online.
