# Supplementary material for: Stenotrophomonas maltophilia: A Gram-Negative Bacterium Useful for Transformations of Flavanone and Chalcone
Source: Molecules. 2017 Oct 27;22(11):1830. doi: 10.3390/molecules22111830 (PMC6150369; doi:10.3390/molecules22111830)

# Supplementary Data

## *Stenotrophomonas maltophilia: a Gram-negative bacterium useful for transformations of flavanone and chalcone*

**Edyta Kostrzewa-Susłow [1,*], Monika Dymarska [1], Urszula Guzik [2], Danuta Wojcieszyńska [2] and Tomasz Janeczko [1]**

[1] Department of Chemistry, Faculty of Biotechnology and Food Science, Wrocław University of Environmental and Life Sciences, Norwida 25, 50-375 Wrocław, Poland
[2] Department of Biochemistry, Faculty of Biology and Environmental Protection, University of Silesia in Katowice, Jagiellonska 28, 40-032 Katowice, Poland
* Correspondence: ekostrzew@gmail.com; Tel.: +48-71-320-5195

**Content**

Figure S1. $^{1}$H NMR spectrum of 7-methoxyflavanone (**1**) (THF-$d_8$, 600 MHz)

Figure S2. $^{13}$C NMR spectrum of 7-methoxyflavanone (**1**) (THF-$d_8$, 151 MHz)

Figure S3. $^{1}$H NMR spectrum of 5, 7-dimethoxyflavanone (**2**) (THF-$d_8$, 600 MHz)

Figure S4. $^{13}$C NMR spectrum of 5, 7-dimethoxyflavanone (**2**) (THF-$d_8$, 151 MHz)

Figure S5. $^{1}$H NMR spectrum of 2′-hydroxy-3-methoxychalcone (**3**) ($CDCl_3$, 600 MHz)

Figure S6. $^{13}$C NMR spectrum of 2′-hydroxy-3-methoxychalcone (**3**) ($CDCl_3$, 151 MHz)

Figure S7. $^{1}$H NMR spectrum of 2′-hydroxy-4′-methoxychalcone (**4**) ($CDCl_3$, 600 MHz)

Figure S8. $^{13}$H NMR spectrum of 2′-hydroxy-4′-methoxychalcone (**4**) ($CDCl_3$, 151 MHz)

Figure S9. $^{1}$H NMR spectrum of 2′-hydroxy-4′-methoxydihydrochalcone (**5**) ($CDCl_3$, 600 MHz**)**

Figure S10. $^{13}$C NMR spectrum of 2′-hydroxy-4′-methoxydihydrochalcone (**5**) ($CDCl_3$, 151 MHz)

Figure S11. $^{1}$H NMR spectrum of 3, 7, 8-trihydroxyflavone (**6**) ($CDCl_3$, 600 MHz)

Figure S12. $^{13}$C NMR spectrum of 3, 7, 8-trihydroxyflavone (**6**) ($CDCl_3$, 151 MHz)

Figure S13. $^{1}$H NMR spectrum of 7-hydroxyflavanone (**7**) (THF, 600 MHz)

Figure S14. $^{13}$C NMR spectrum of 7-hydroxyflavanone (**7**) (THF, 151 MHz)

Figure S15. $^{1}$H NMR spectrum of 2′-hydroxy-4′, 6′-dimethoxychalcone (**8**) ($CDCl_3$, 600 MHz)

Figure S16. $^{13}$C NMR spectrum of 2′-hydroxy-4′, 6′-dimethoxychalcone (**8**) ($CDCl_3$, 151 MHz)

Figure S17. $^{1}$H NMR spectrum of 2′-hydroxy-4′, 6′-dimethoxydihydrochalcone (**9**) ($CDCl_3$, 600 MHz)

Figure S18. $^{13}$C NMR spectrum of 2′-hydroxy-4′, 6′-dimethoxydihydrochalcone (**9**) ($CDCl_3$, 151 MHz)

Figure S19. $^{1}$H NMR spectrum of 2′-hydroxy-3-methoxydihydrochalcone (**10**) ($CDCl_3$, 600 MHz)

Figure S20. $^{13}$C NMR spectrum of 2′-hydroxy-3-methoxydihydrochalcone (**10**) ($CDCl_3$, 151 MHz)

Figure S21. $^{1}$H NMR spectrum of 3′-methoxyflavanone (**11**) ($CDCl_3$, 600 MHz)

Figure S22. $^{13}$C NMR spectrum of 3′-methoxyflavanone (**11**) ($CDCl_3$, 151 MHz)

Figure S1. $^{1}$H NMR spectrum of 7-methoxyflavanone (**1**) (THF-d8, 600 MHz)

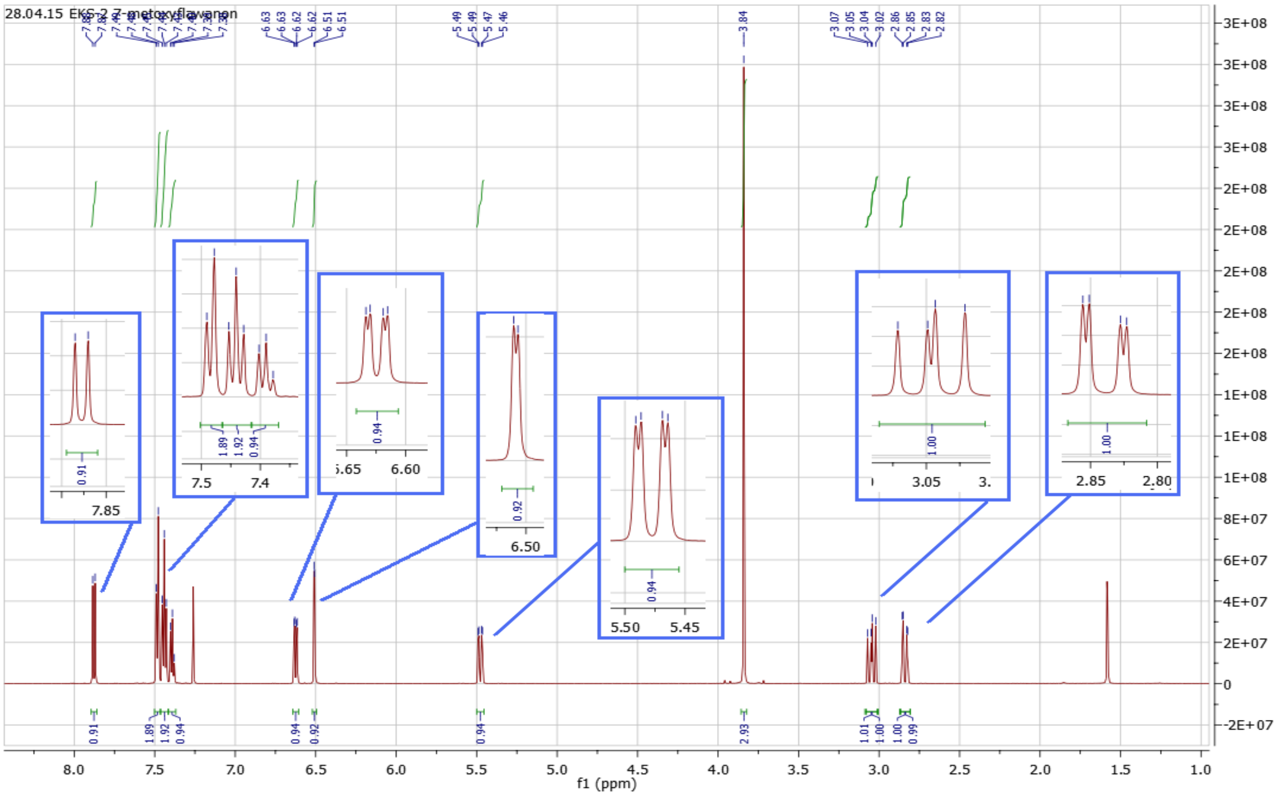


Figure S2. $^{13}$C NMR spectrum of 7-methoxyflavanone (**1**) (THF-d8, 151 MHz)

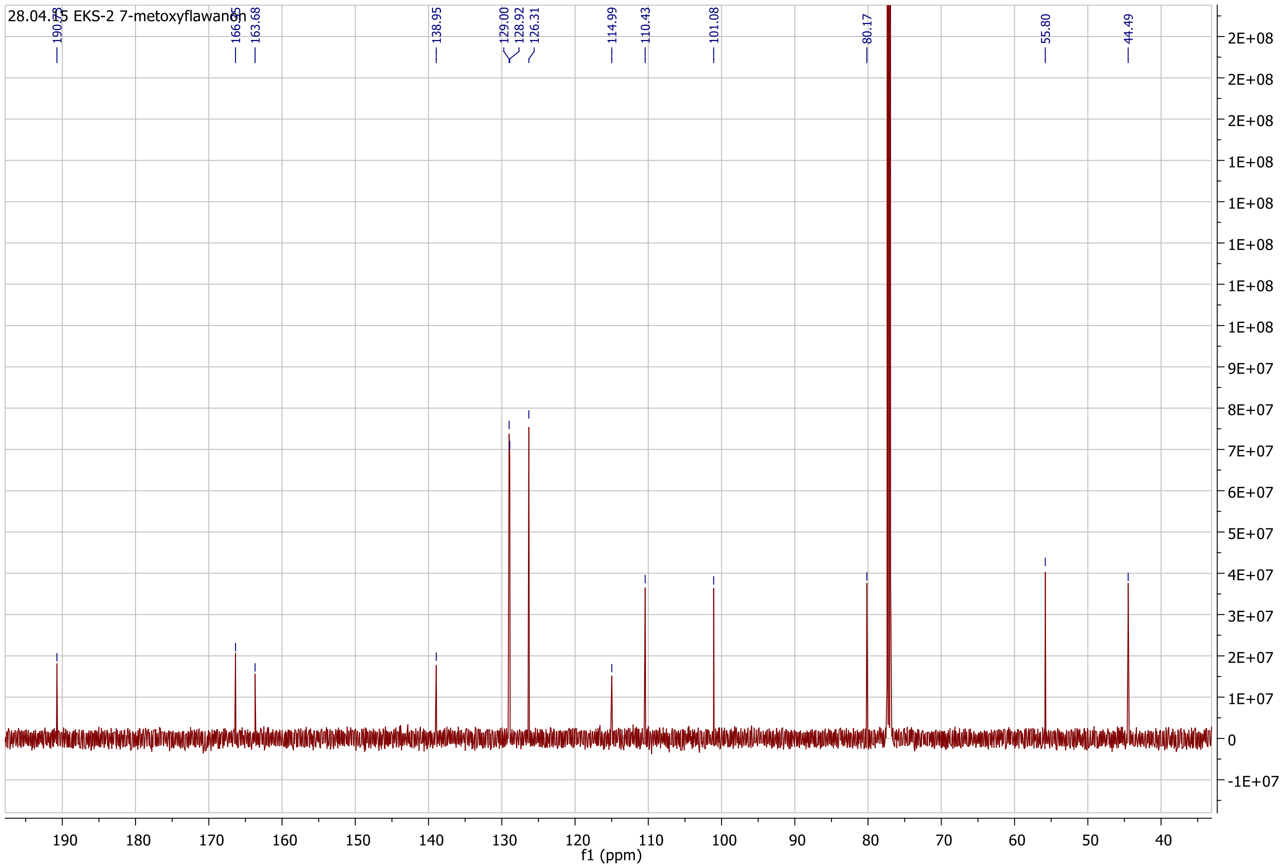

Figure S3. $^{1}$H NMR spectrum of 5, 7-dimethoxyflavanone (**2**) (THF-d8, 600 MHz)

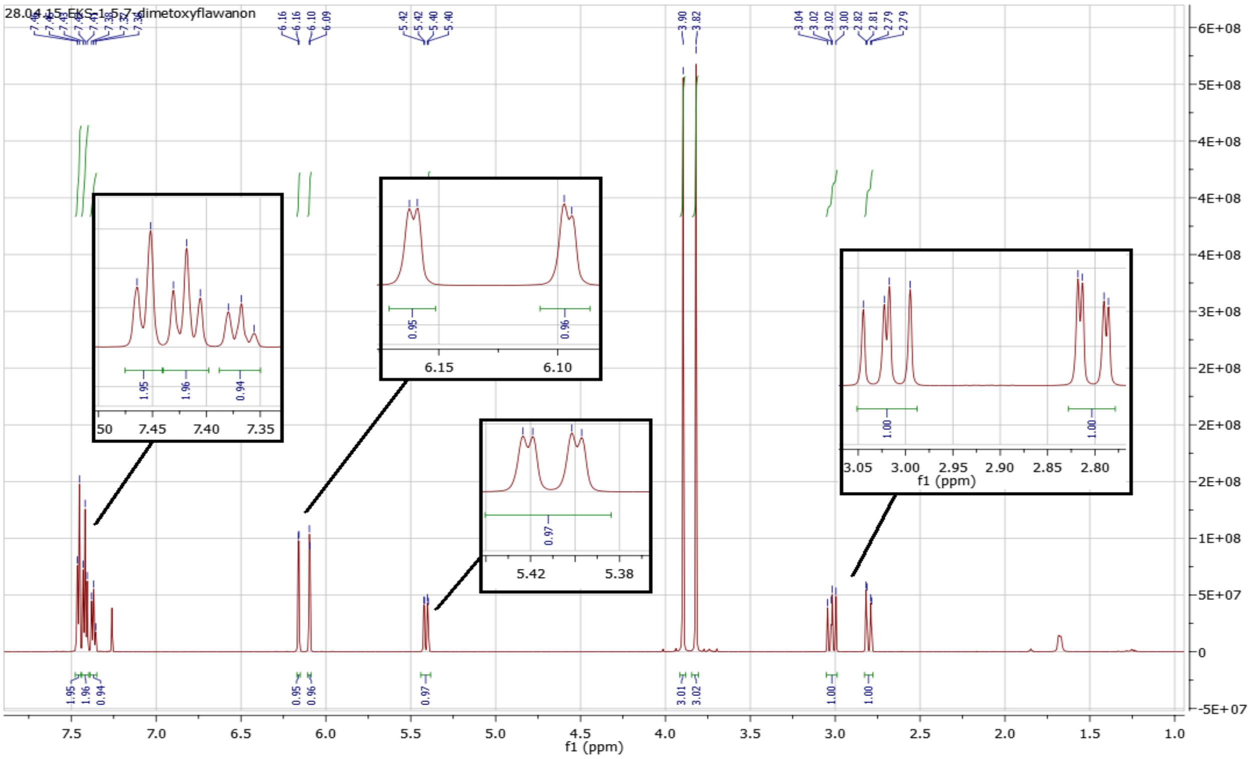


Figure S4. $^{13}$C NMR spectrum of 5, 7-dimethoxyflavanone (**2**) (THF-d8, 151 MHz)

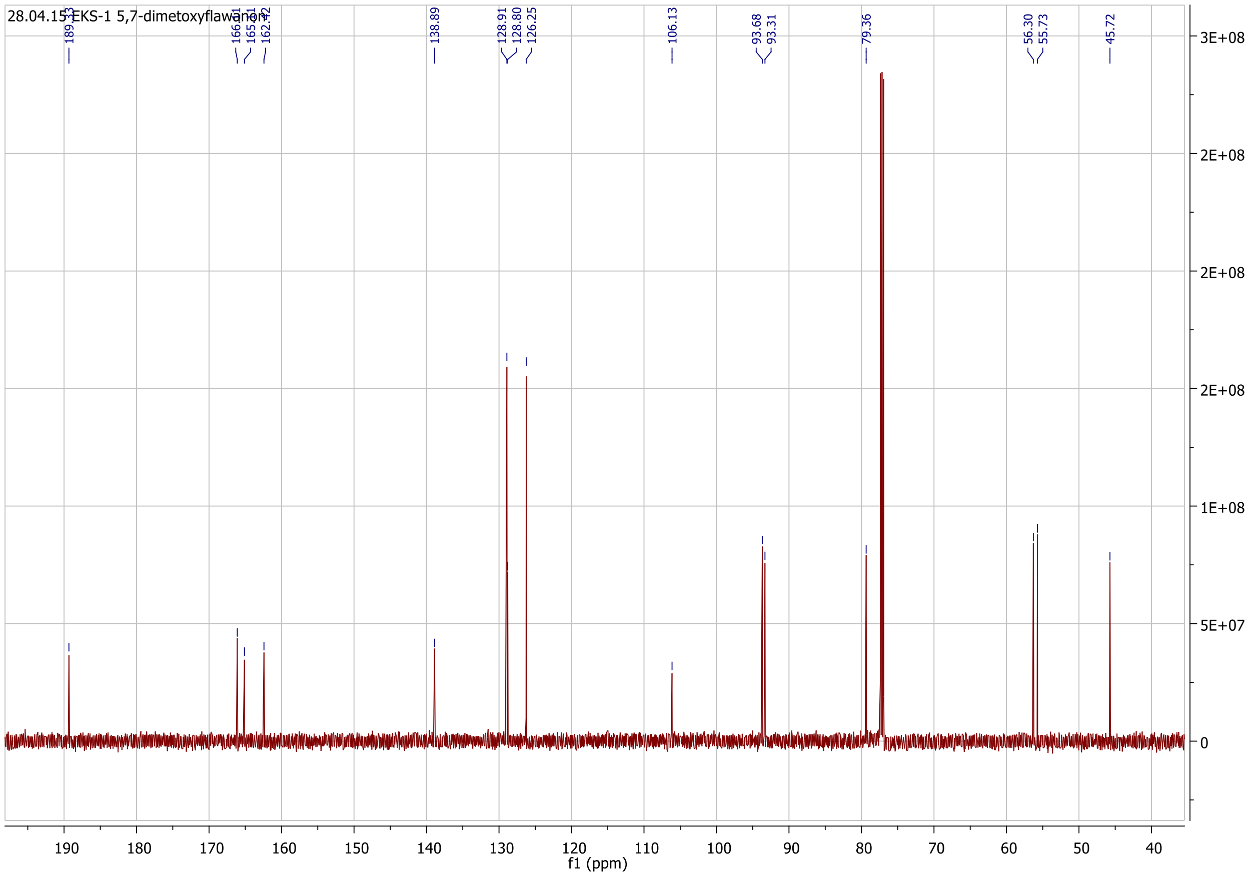

Figure S5. $^{1}$H NMR spectrum of 2′-hydroxy-3-methoxychalcone (**3**) ($CDCl_3$, 600 MHz)

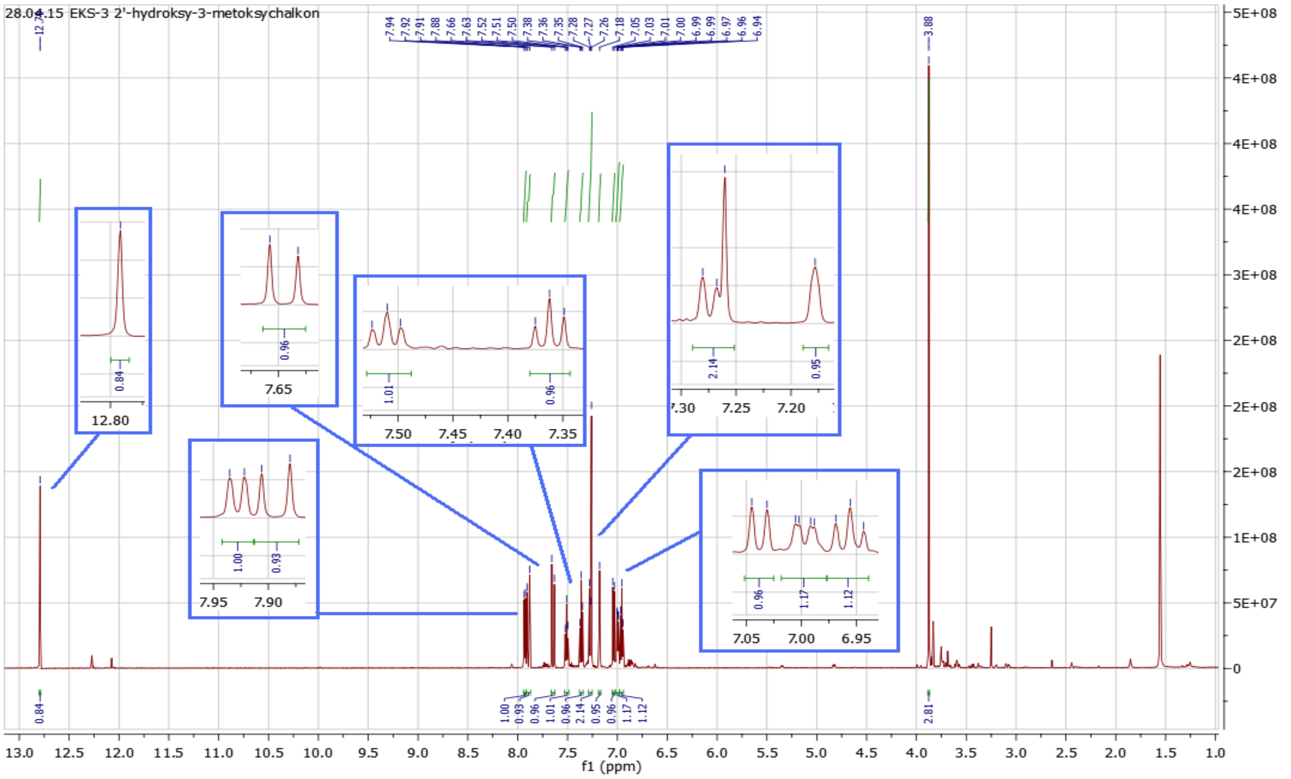


Figure S6. $^{13}$C NMR spectrum of 2′-hydroxy-3-methoxychalcone (**3**) ($CDCl_3$, 151 MHz)

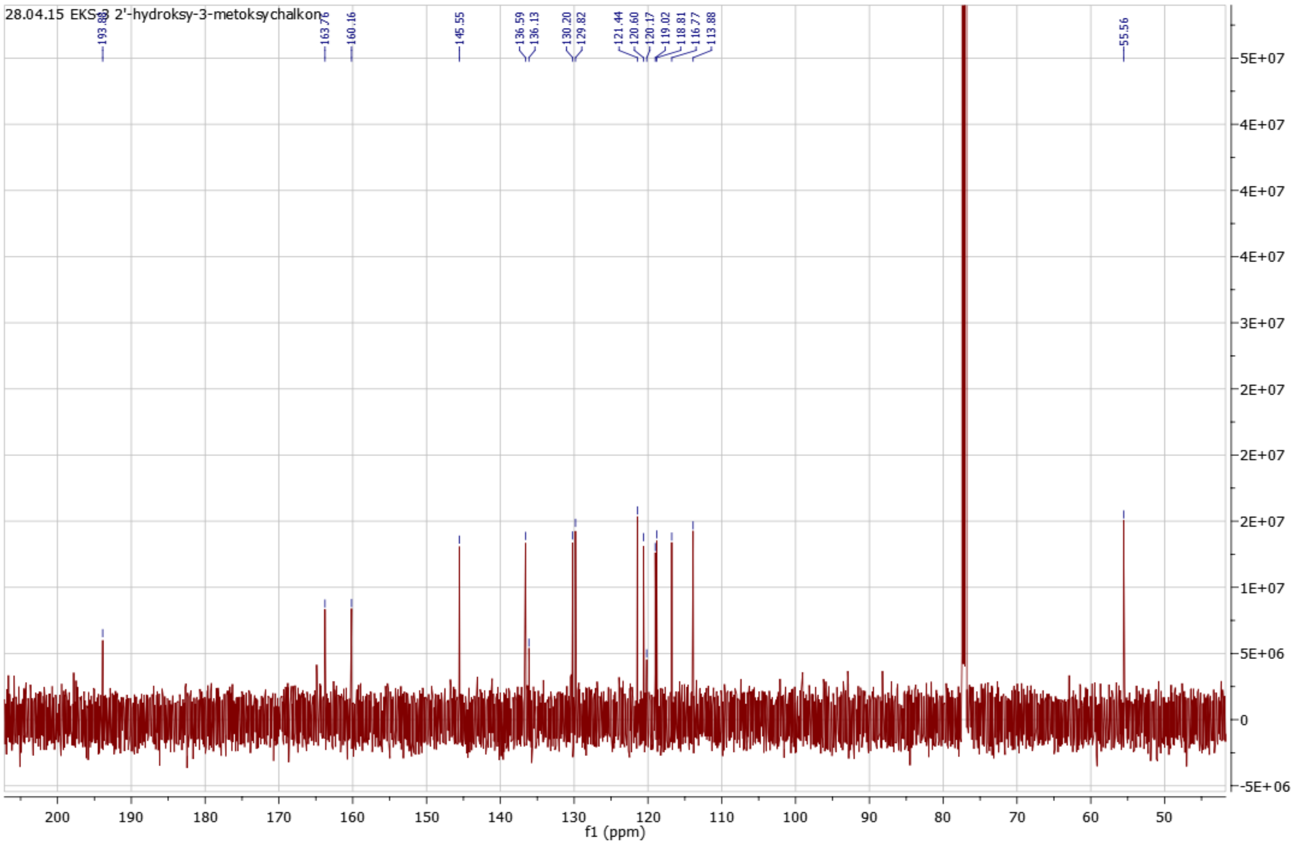

Figure S7. $^{1}$H NMR spectrum of 2′-hydroxy-4′-methoxychalcone (**4**) ($CDCl_3$, 600 MHz)

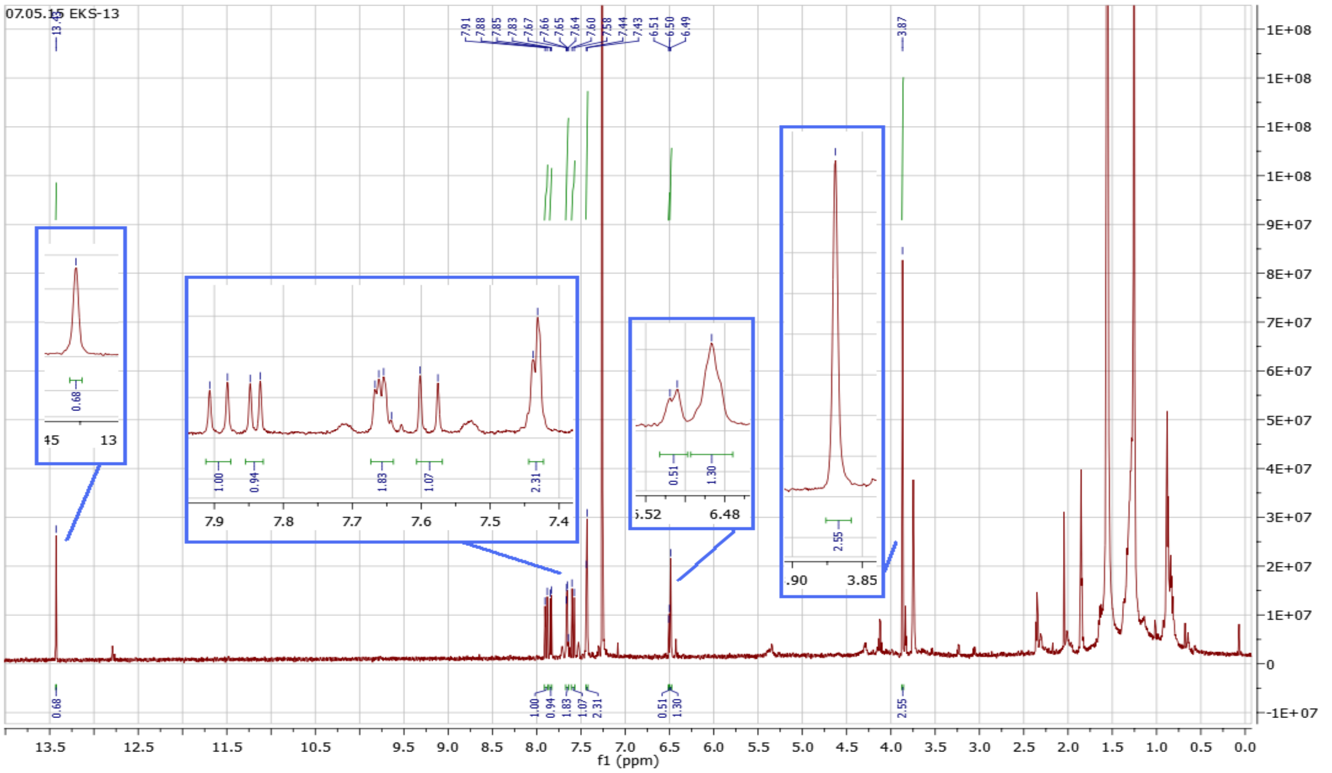


Figure S8. [13]H NMR spectrum of 2′-hydroxy-4′-methoxychalcone (**4**) ($CDCl_3$, 151 MHz)

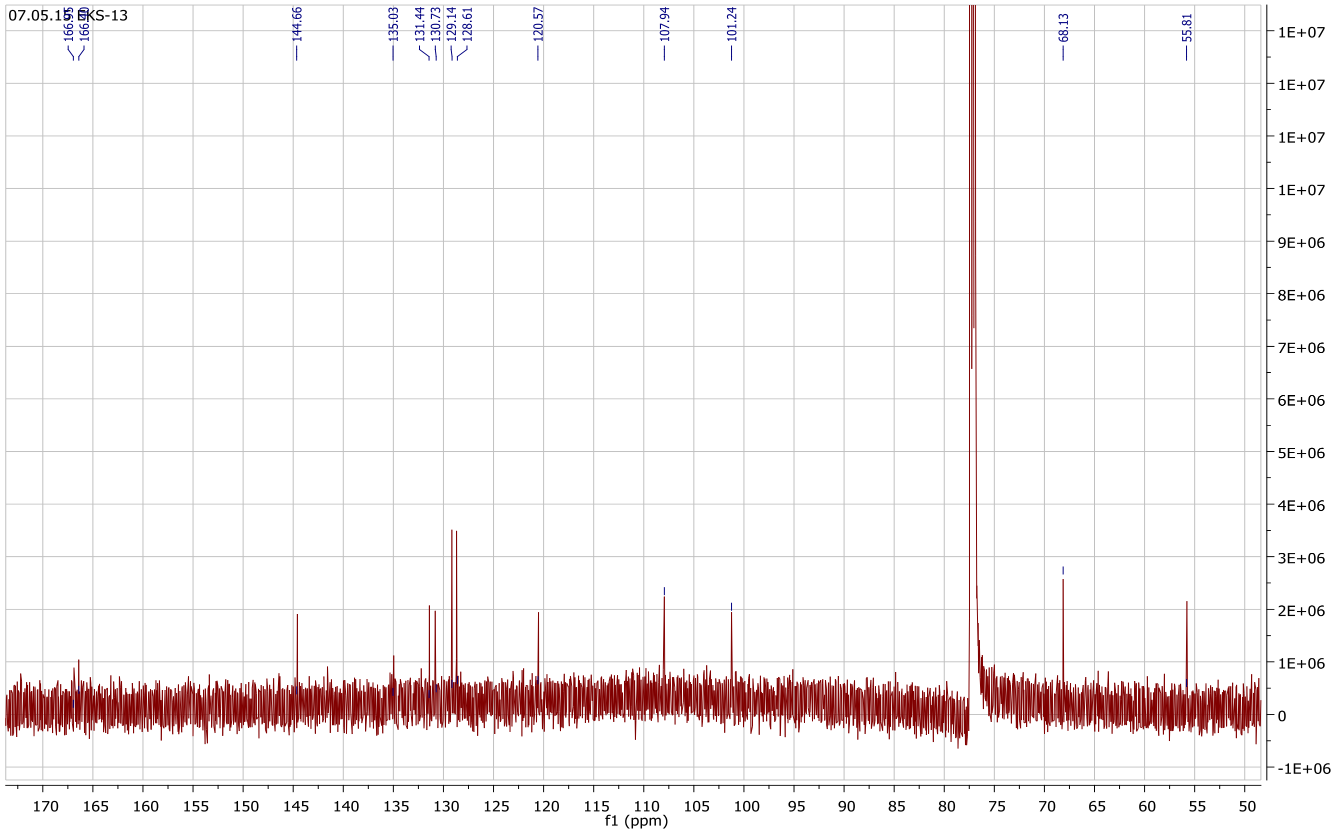

Figure S9. $^{1}$H NMR spectrum of 2′-hydroxy-4′-methoxydihydrochalcone (**5**) ($CDCl_3$, 600 MHz)

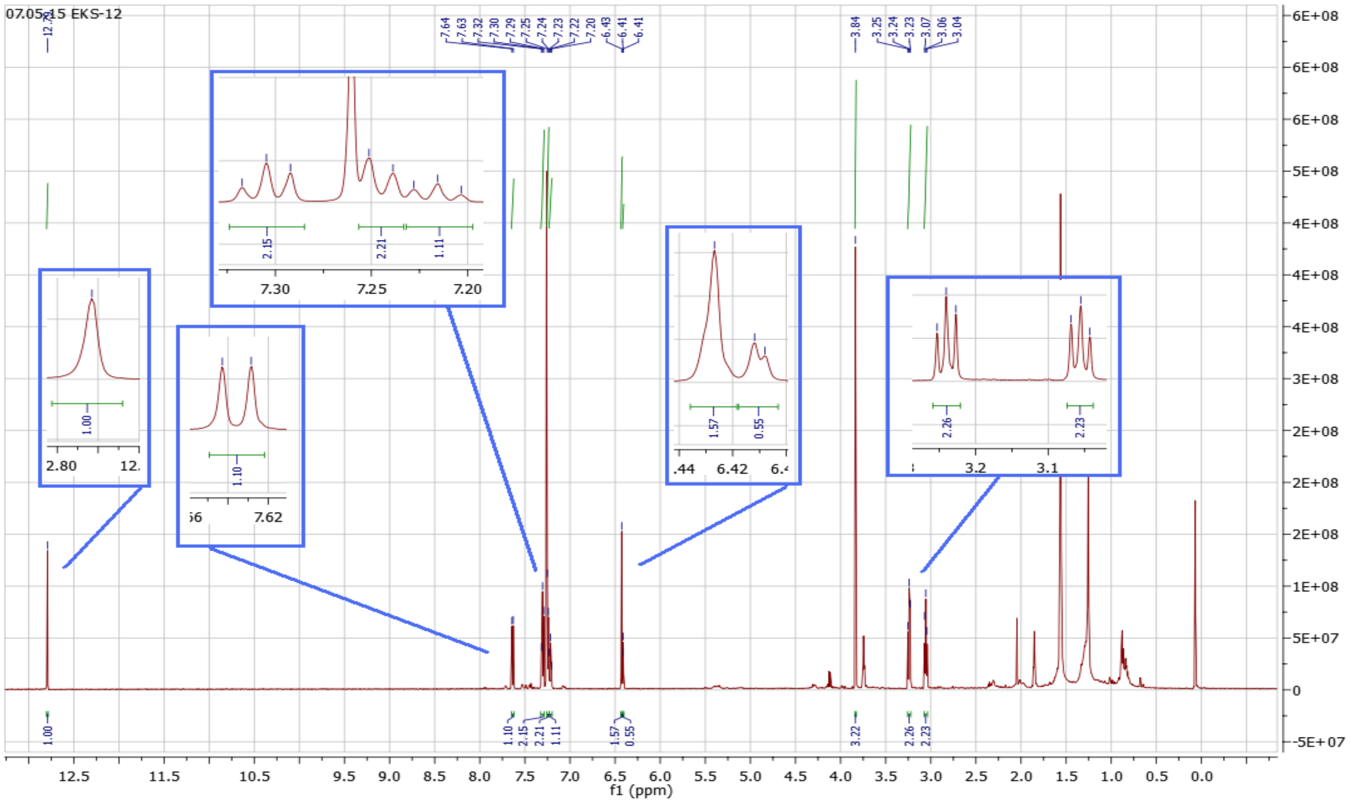

Figure S10. $^{13}$C NMR spectrum of 2′-hydroxy-4′-methoxydihydrochalcone (**5**) ($CDCl_3$, 151 MHz)

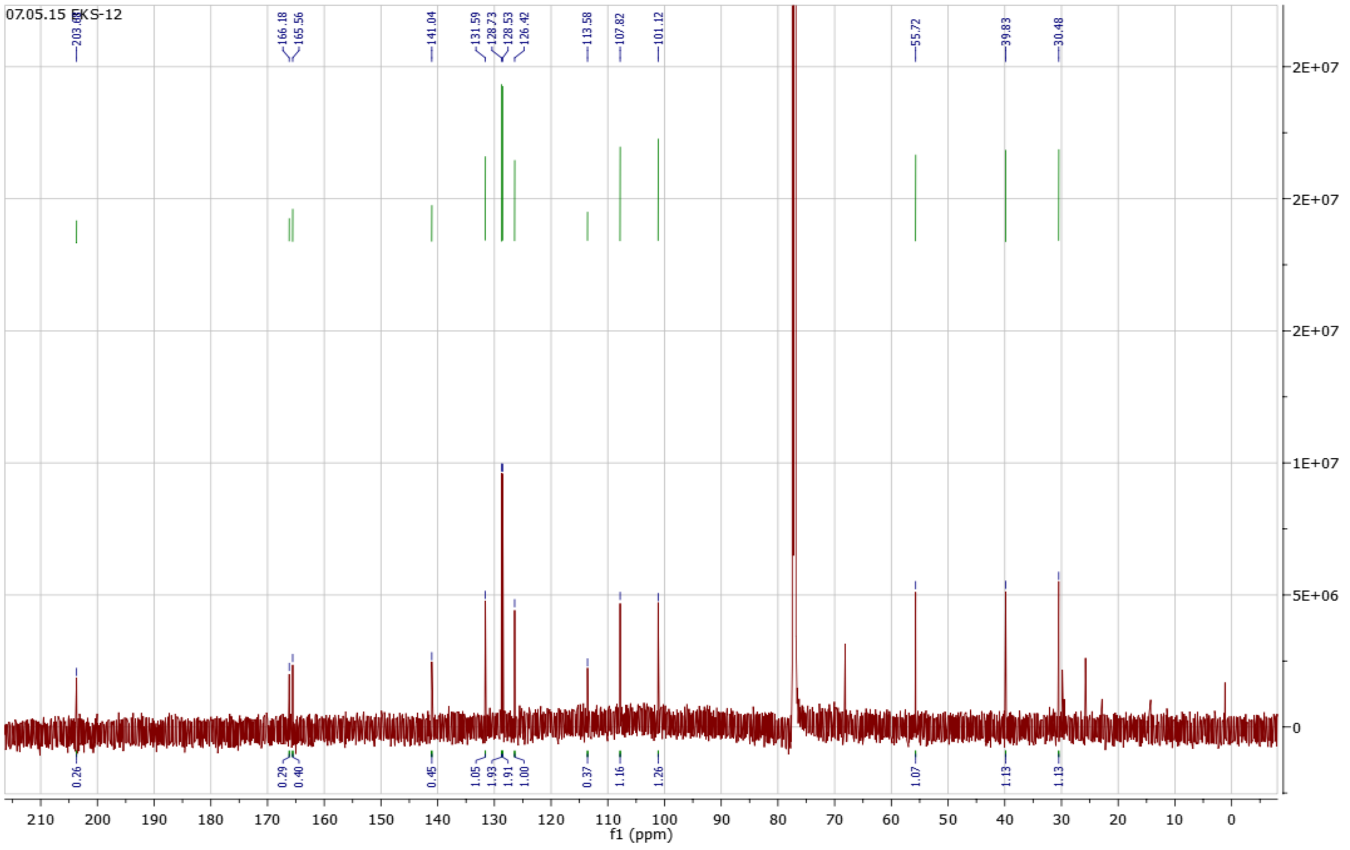

Figure S11. $^{1}$H NMR spectrum of 3,7,8-trihydroxyflavone (**6**) ($CDCl_3$, 600 MHz)

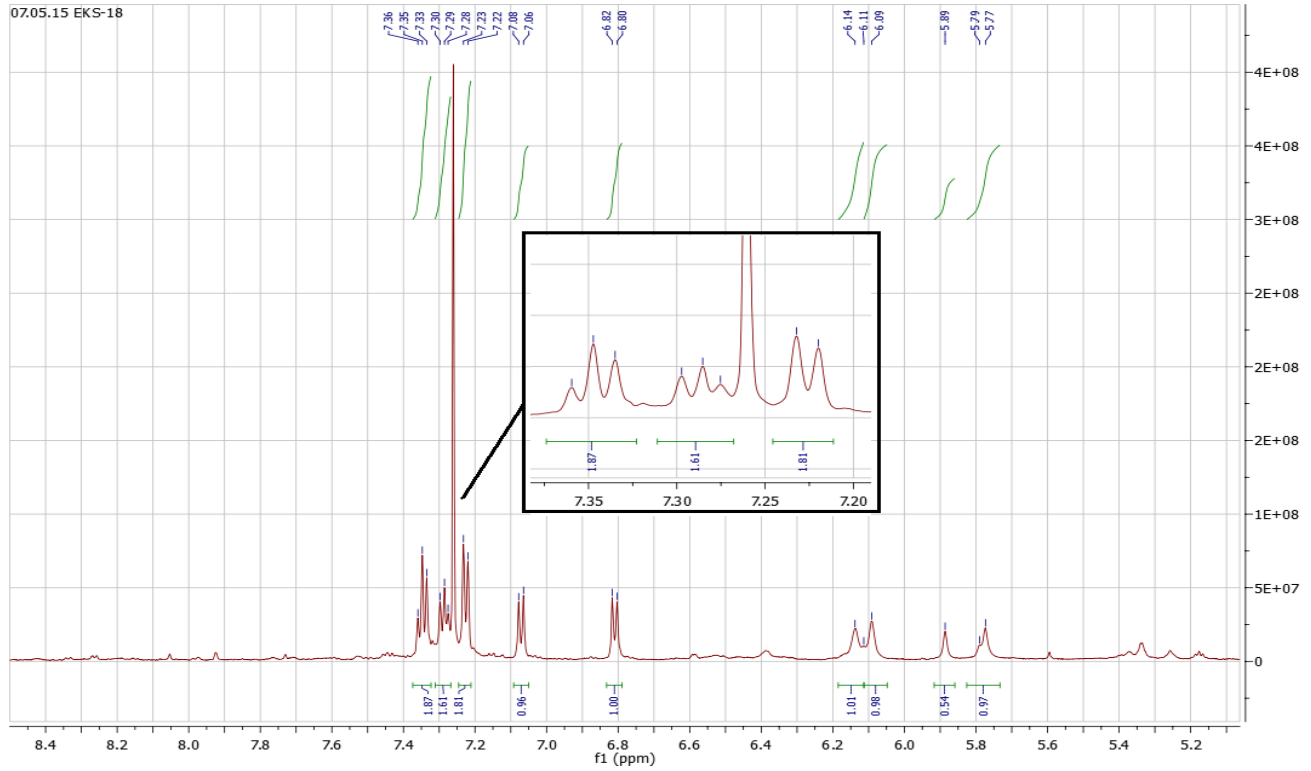

Figure S12. $^{13}$C NMR spectrum of 3,7,8-trihydroxyflavone (**6**) ($CDCl_3$, 151 MHz)

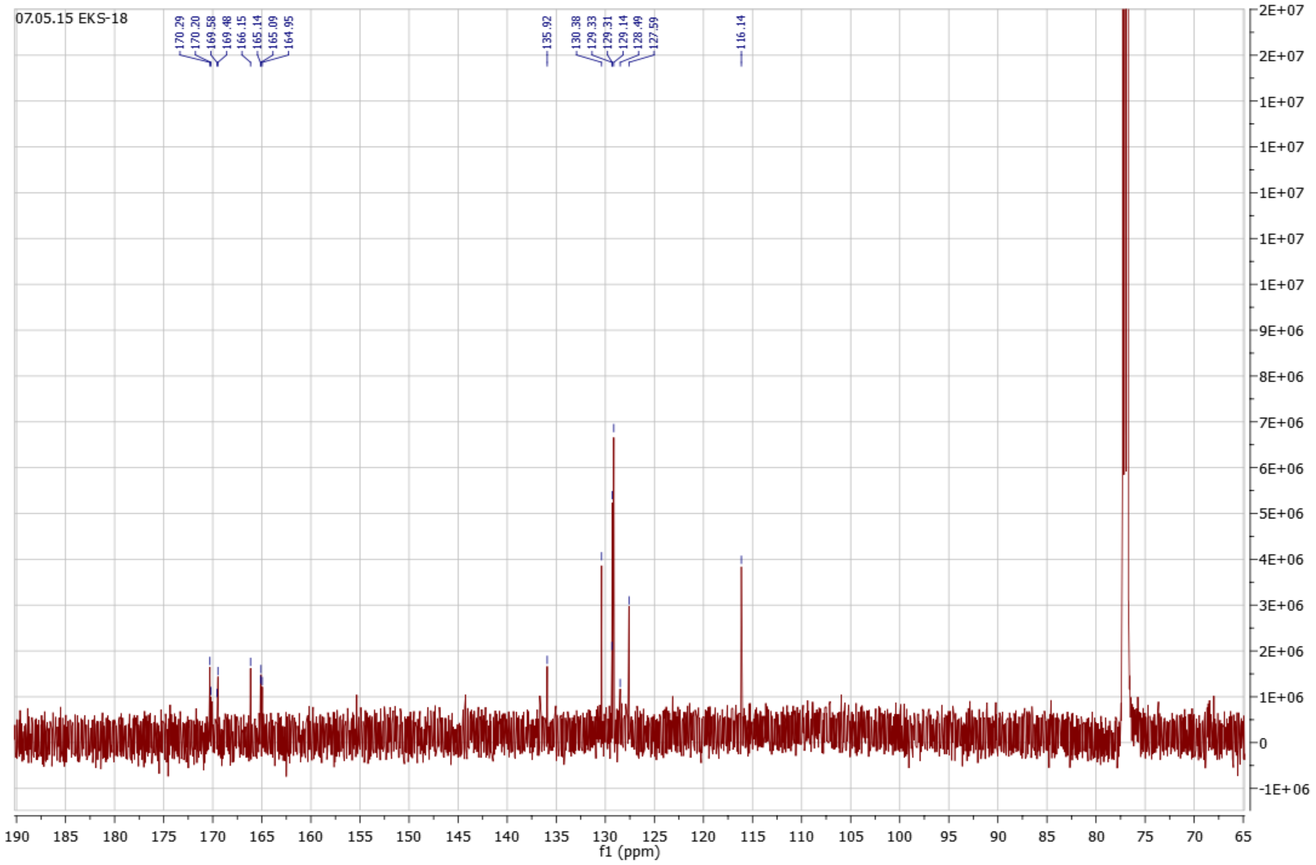

Figure S13. $^{1}$H NMR spectrum of 7-hydroxyflavanone (**7**) (THF, 600 MHz)

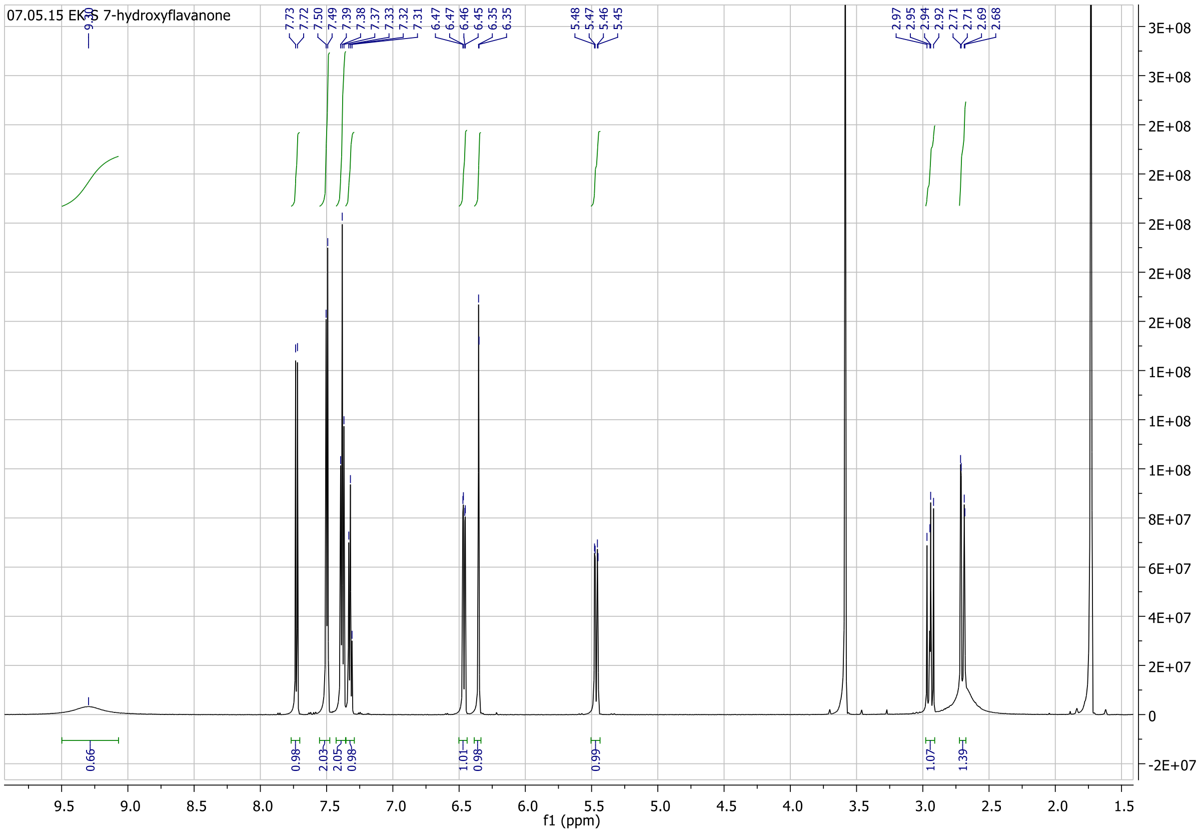


Figure S14. $^{13}$C NMR spectrum of 7-hydroxyflavanone (**7**) (THF, 151 MHz)

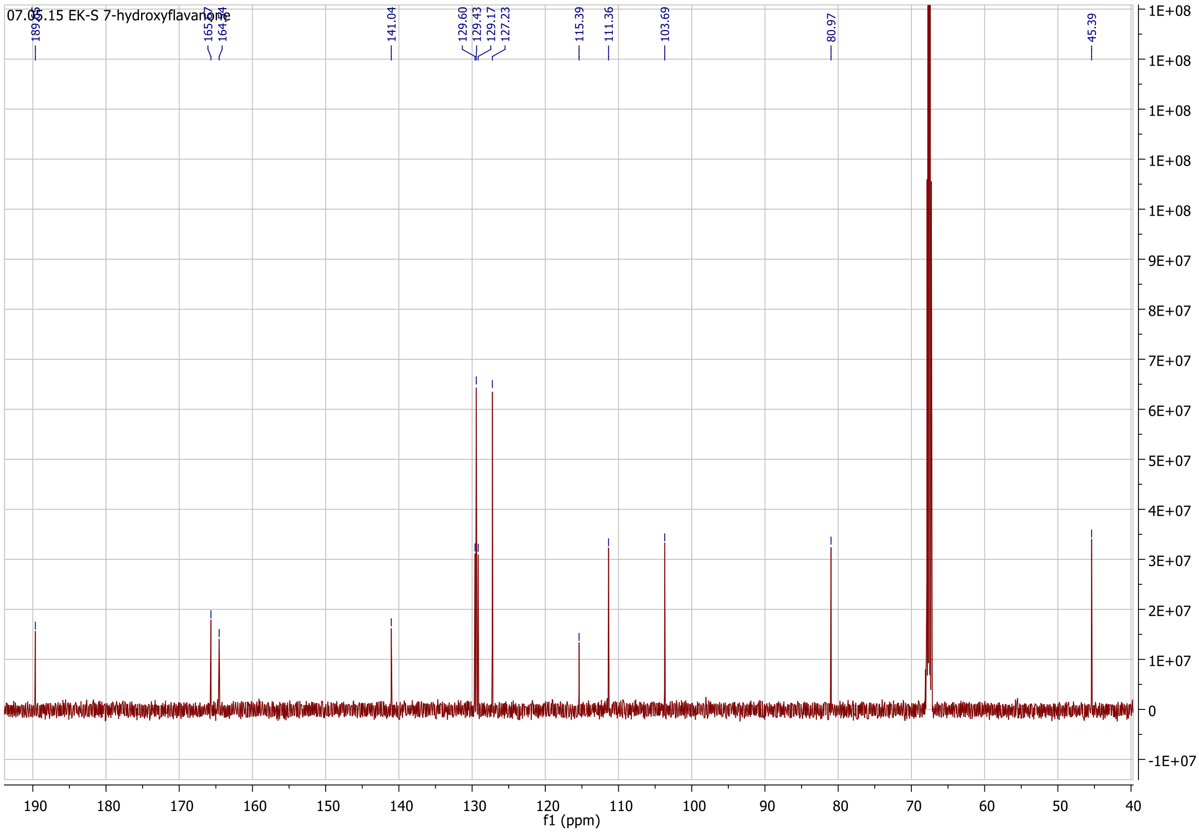

Figure S15. $^{1}$H NMR spectrum of 2′-hydroxy-4′, 6′-dimethoxychalcone (**8**) ($CDCl_3$, 600 MHz)

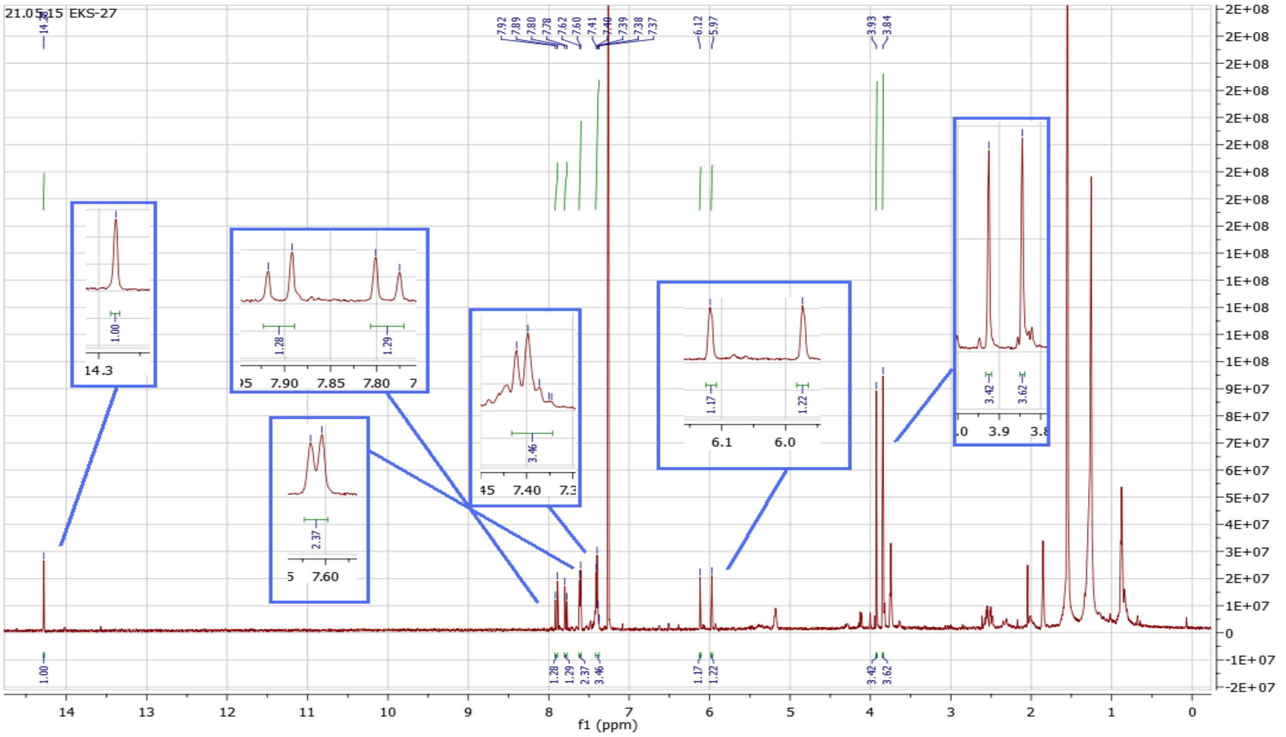


Figure S16. $^{13}$C NMR spectrum of 2′-hydroxy-4′, 6′-dimethoxychalcone (**8**) ($CDCl_3$, 151 MHz)

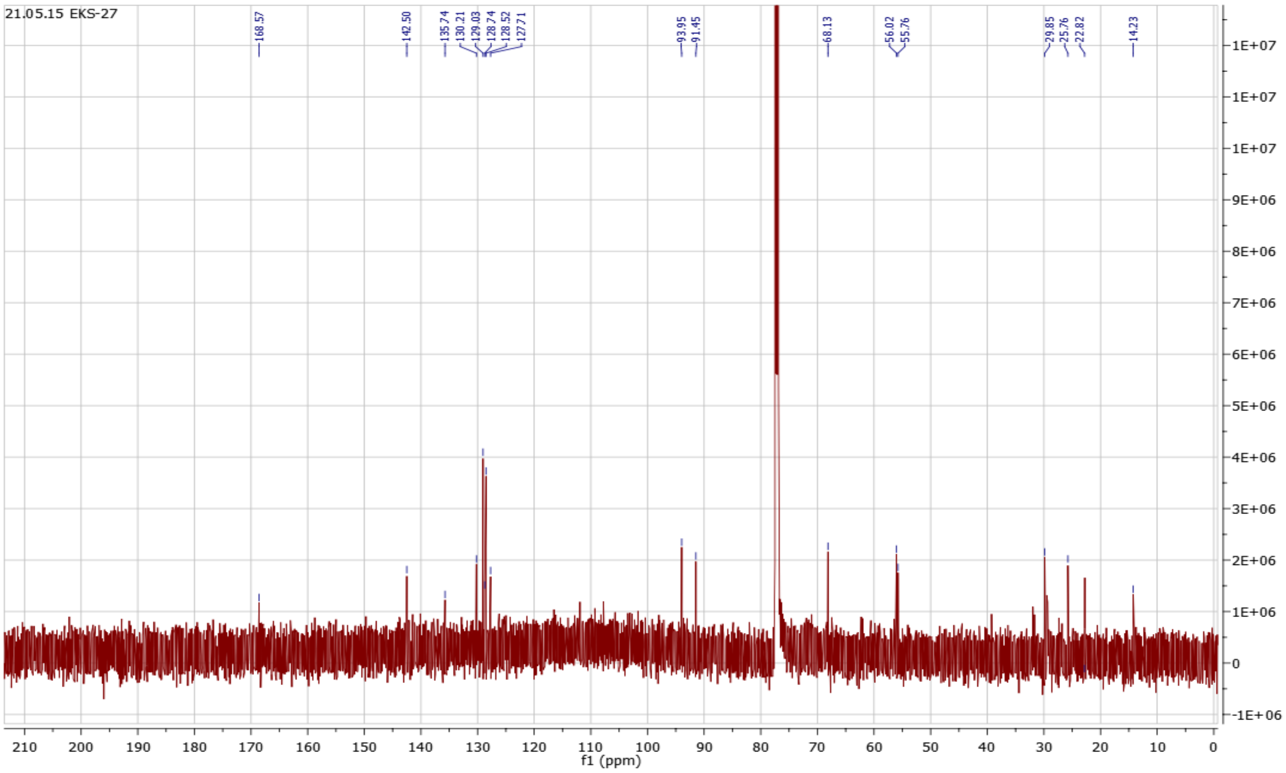

Figure S17. $^{1}$H NMR spectrum of 2′-hydroxy-4′,6′-dimethoxydihydrochalcone (**9**) ($CDCl_3$, 600 MHz)

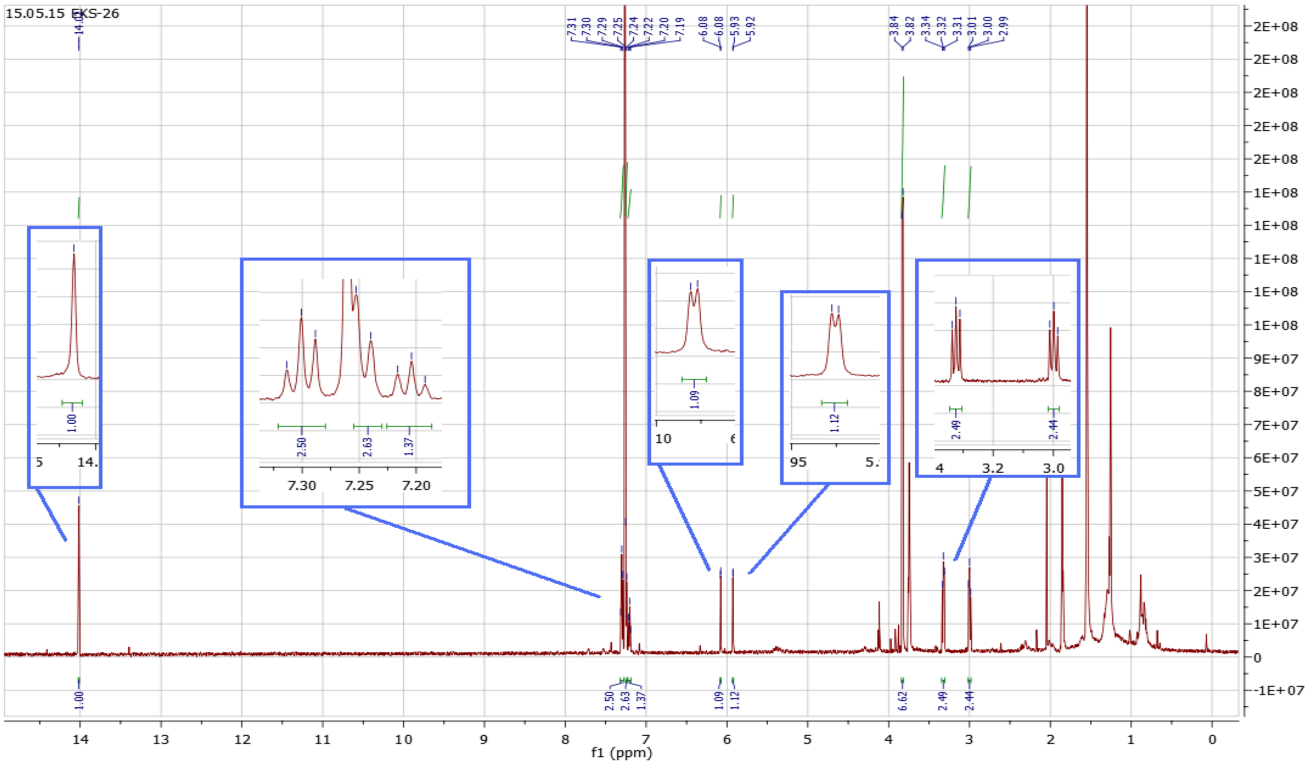


Figure S18. $^{13}$C NMR spectrum of 2′-hydroxy-4′,6′-dimethoxydihydrochalcone (**9**) ($CDCl_3$, 151 MHz)

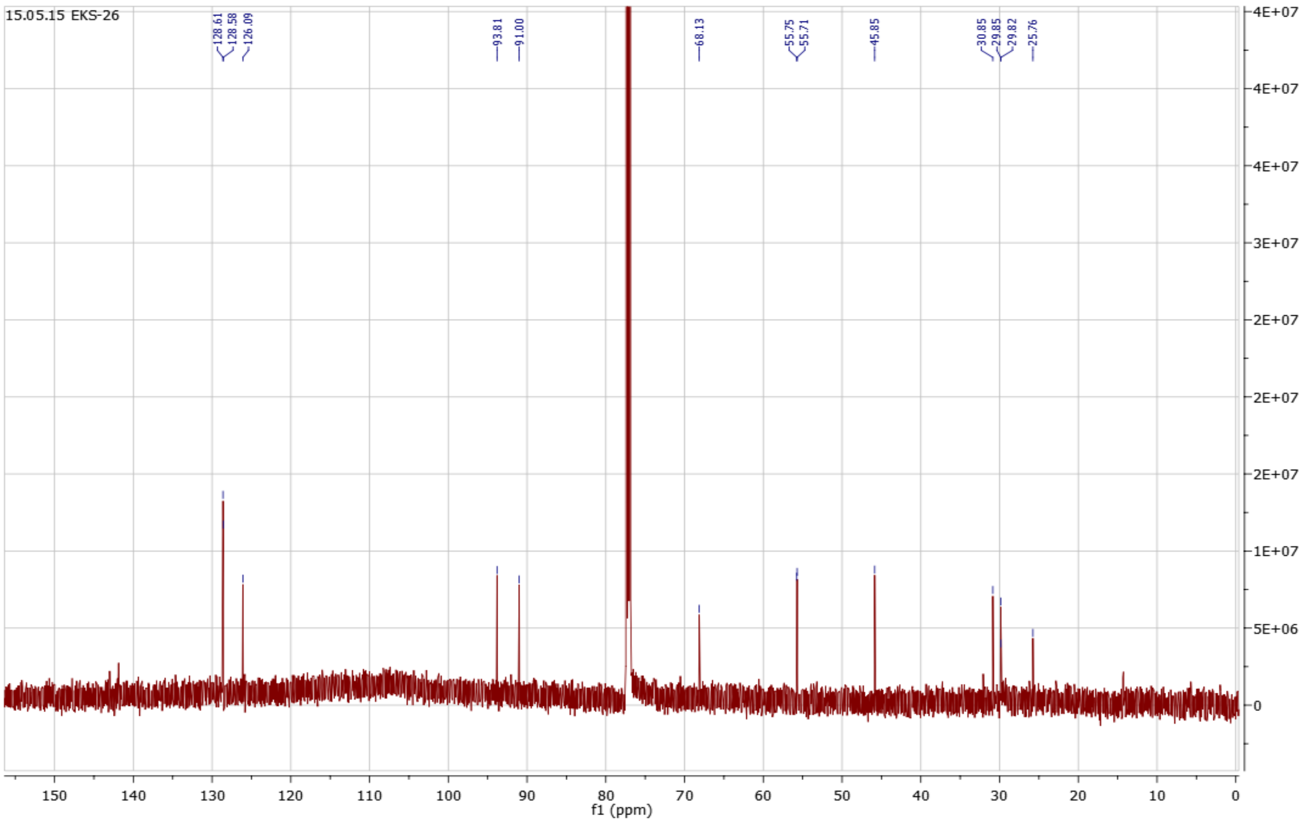

Figure S19. $^{1}H$ NMR spectrum of 2′-hydroxy-3-methoxydihydrochalcone (**10**) ($CDCl_3$, 600 MHz)

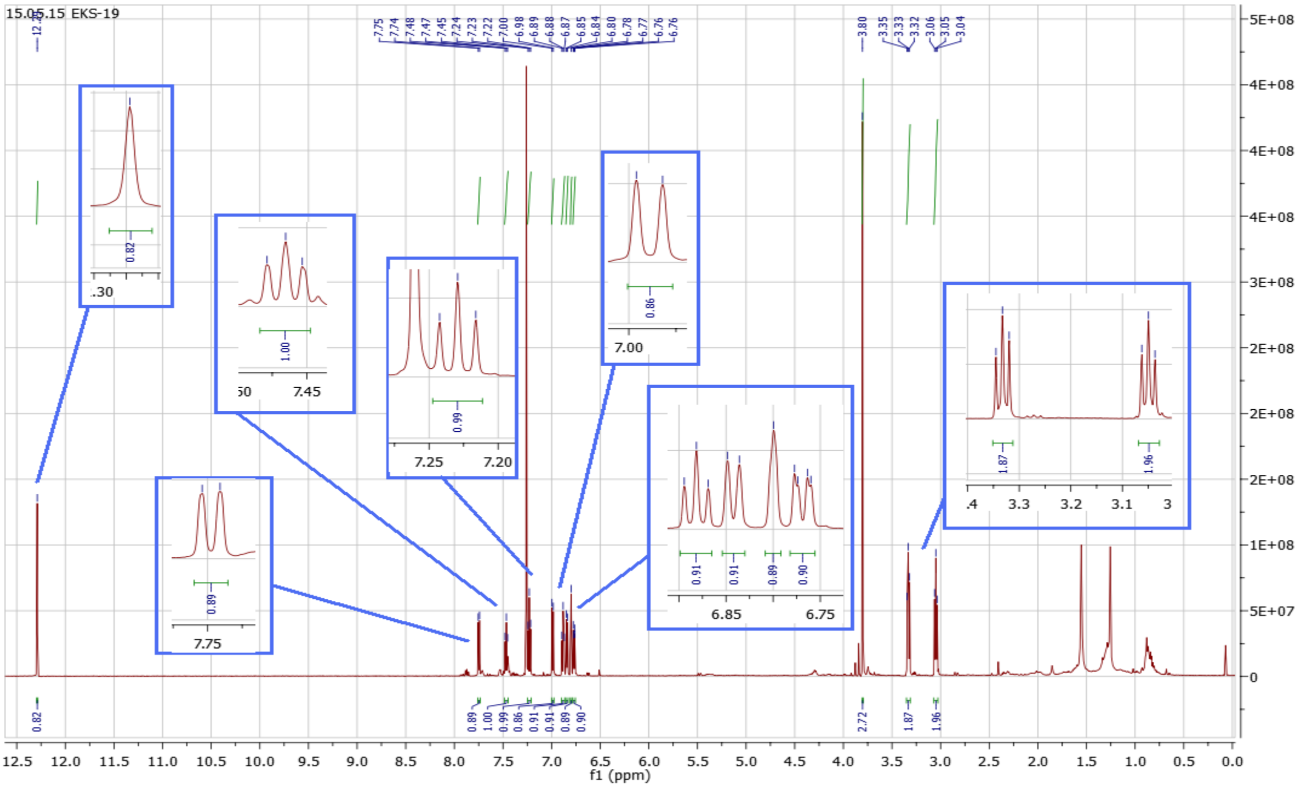


Figure S20. $^{13}C$ NMR spectrum of 2′-hydroxy-3-methoxydihydrochalcone (**10**) ($CDCl_3$, 151 MHz)

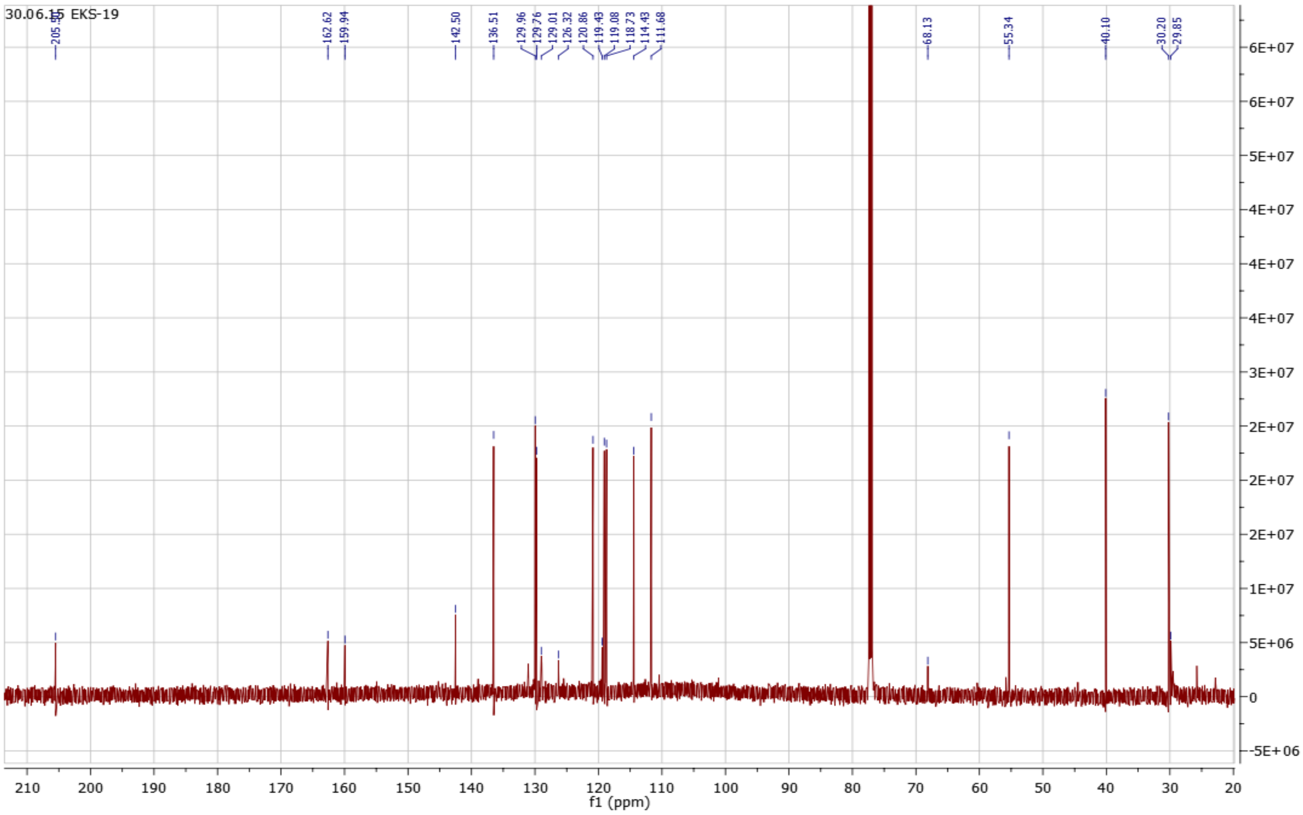

Figure S21. $^{1}$H NMR spectrum of 3′-methoxyflavanone (**11**) ($CDCl_3$, 600 MHz)

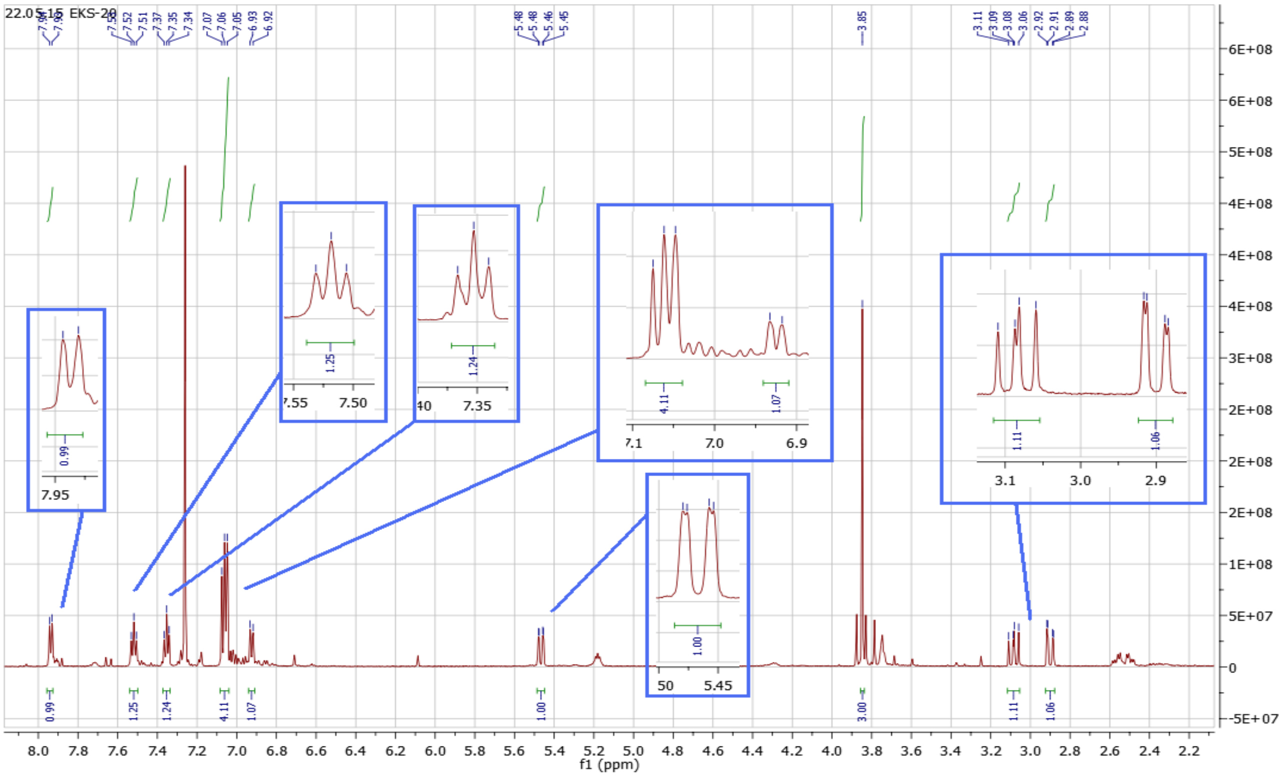

Figure S22. $^{13}$C NMR spectrum of 3′-methoxyflavanone (**11**) ($CDCl_3$, 151 MHz)

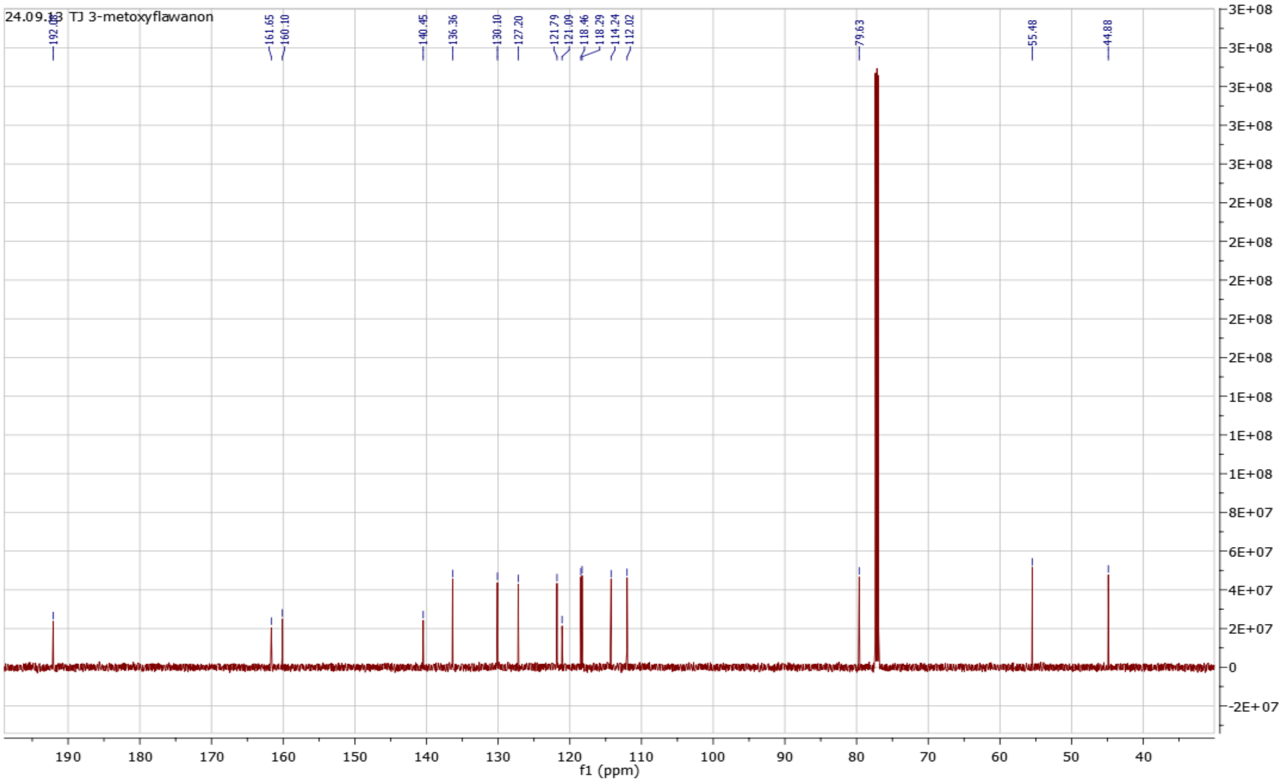

Supplement: Supplementary file 1 [file molecules-22-01830-s001.pdf]
